# Luminosity determination using Z boson production at the CMS experiment

**DOI:** 10.1140/epjc/s10052-023-12268-2

**Published:** 2024-01-10

**Authors:** CMS Collaboration, A. Hayrapetyan, A. Tumasyan, W. Adam, J. W. Andrejkovic, T. Bergauer, S. Chatterjee, K. Damanakis, M. Dragicevic, A. Escalante Del Valle, P.S. Hussain, M. Jeitler, N. Krammer, D. Liko, I. Mikulec, J. Schieck, R. Schöfbeck, D. Schwarz, M. Sonawane, S. Templ, W. Waltenberger, C.-E. Wulz, M.R. Darwish, T. Janssen, P. Van Mechelen, E.S. Bols, J. D’Hondt, S. Dansana, A. De Moor, M. Delcourt, H. El Faham, S. Lowette, I. Makarenko, A. Morton, D. Müller, A.R. Sahasransu, S. Tavernier, M. Tytgat, S. Van Putte, D. Vannerom, B. Clerbaux, G. De Lentdecker, L. Favart, D. Hohov, J. Jaramillo, A. Khalilzadeh, K. Lee, M. Mahdavikhorrami, A. Malara, S. Paredes, L. Pétré, N. Postiau, L. Thomas, M. Vanden Bemden, C. Vander Velde, P. Vanlaer, M. De Coen, D. Dobur, J. Knolle, L. Lambrecht, G. Mestdach, C. Rendón, A. Samalan, K. Skovpen, N. Van Den Bossche, L. Wezenbeek, A. Benecke, G. Bruno, C. Caputo, C. Delaere, I.S. Donertas, A. Giammanco, K. Jaffel, Sa. Jain, V. Lemaitre, J. Lidrych, P. Mastrapasqua, K. Mondal, T.T. Tran, S. Wertz, G.A. Alves, E. Coelho, C. Hensel, T. Menezes De Oliveira, A. Moraes, P. Rebello Teles, M. Soeiro, W.L. Aldá Júnior, M. Alves Gallo Pereira, M. Barroso Ferreira Filho, H. Brandao Malbouisson, W. Carvalho, J. Chinellato, E.M. Da Costa, G.G. Da Silveira, D. De Jesus Damiao, S. Fonseca De Souza, J. Martins, C. Mora Herrera, K. Mota Amarilo, L. Mundim, H. Nogima, A. Santoro, S.M. Silva Do Amaral, A. Sznajder, M. Thiel, A. Vilela Pereira, C.A. Bernardes, L. Calligaris, T.R. Fernandez Perez Tomei, E.M. Gregores, P.G. Mercadante, S.F. Novaes, B. Orzari, Sandra S. Padula, A. Aleksandrov, G. Antchev, R. Hadjiiska, P. Iaydjiev, M. Misheva, M. Shopova, G. Sultanov, A. Dimitrov, T. Ivanov, L. Litov, B. Pavlov, P. Petkov, A. Petrov, E. Shumka, S. Keshri, S. Thakur, T. Cheng, Q. Guo, T. Javaid, M. Mittal, L. Yuan, G. Bauer, Z. Hu, K. Yi, G.M. Chen, H.S. Chen, M. Chen, F. Iemmi, C. H. Jiang, A. Kapoor, H. Liao, Z.-A. Liu, F. Monti, R. Sharma, J. N. Song, J. Tao, J. Wang, H. Zhang, A. Agapitos, Y. Ban, A. Levin, C. Li, Q. Li, X. Lyu, Y. Mao, S.J. Qian, X. Sun, D. Wang, H. Yang, C. Zhou, Z. You, N. Lu, X. Gao, D. Leggat, H. Okawa, Y. Zhang, Z. Lin, C. Lu, M. Xiao, C. Avila, D. A. Barbosa Trujillo, A. Cabrera, C. Florez, J. Fraga, J. A. Reyes Vega, J. Mejia Guisao, F. Ramirez, M. Rodriguez, J.D. Ruiz Alvarez, D. Giljanovic, N. Godinovic, D. Lelas, A. Sculac, M. Kovac, T. Sculac, P. Bargassa, V. Brigljevic, B.K. Chitroda, D. Ferencek, S. Mishra, A. Starodumov, T. Susa, A. Attikis, K. Christoforou, S. Konstantinou, J. Mousa, C. Nicolaou, F. Ptochos, P.A. Razis, H. Rykaczewski, H. Saka, A. Stepennov, M. Finger, M. Finger, A. Kveton, E. Ayala, E. Carrera Jarrin, A.A. Abdelalim, E. Salama, M. Abdullah Al-Mashad, M.A. Mahmoud, R.K. Dewanjee, K. Ehataht, M. Kadastik, T. Lange, S. Nandan, C. Nielsen, J. Pata, M. Raidal, L. Tani, C. Veelken, H. Kirschenmann, K. Osterberg, M. Voutilainen, S. Bharthuar, E. Brücken, F. Garcia, J. Havukainen, K.T.S. Kallonen, M.S. Kim, R. Kinnunen, T. Lampén, K. Lassila-Perini, S. Lehti, T. Lindén, M. Lotti, L. Martikainen, M. Myllymäki, M.m. Rantanen, H. Siikonen, E. Tuominen, J. Tuominiemi, P. Luukka, H. Petrow, T. Tuuva, M. Besancon, F. Couderc, M. Dejardin, D. Denegri, J. L. Faure, F. Ferri, S. Ganjour, P. Gras, G. Hamel de Monchenault, V. Lohezic, J. Malcles, J. Rander, A. Rosowsky, M.Ö. Sahin, A. Savoy-Navarro, P. Simkina, M. Titov, C. Baldenegro Barrera, F. Beaudette, A. Buchot Perraguin, P. Busson, A. Cappati, C. Charlot, F. Damas, O. Davignon, G. Falmagne, B.A. Fontana Santos Alves, S. Ghosh, A. Gilbert, R. Granier de Cassagnac, A. Hakimi, B. Harikrishnan, L. Kalipoliti, G. Liu, J. Motta, M. Nguyen, C. Ochando, L. Portales, R. Salerno, U. Sarkar, J.B. Sauvan, Y. Sirois, A. Tarabini, E. Vernazza, A. Zabi, A. Zghiche, J.-L. Agram, J. Andrea, D. Apparu, D. Bloch, J.-M. Brom, E.C. Chabert, C. Collard, S. Falke, U. Goerlach, C. Grimault, R. Haeberle, A.-C. Le Bihan, M.A. Sessini, P. Van Hove, S. Beauceron, B. Blancon, G. Boudoul, N. Chanon, J. Choi, D. Contardo, P. Depasse, C. Dozen, H. El Mamouni, J. Fay, S. Gascon, M. Gouzevitch, C. Greenberg, G. Grenier, B. Ille, I. B. Laktineh, M. Lethuillier, L. Mirabito, S. Perries, M. Vander Donckt, P. Verdier, J. Xiao, D. Chokheli, I. Lomidze, Z. Tsamalaidze, V. Botta, L. Feld, K. Klein, M. Lipinski, D. Meuser, A. Pauls, N. Röwert, M. Teroerde, S. Diekmann, A. Dodonova, N. Eich, D. Eliseev, F. Engelke, M. Erdmann, P. Fackeldey, B. Fischer, T. Hebbeker, K. Hoepfner, F. Ivone, A. Jung, M.y. Lee, L. Mastrolorenzo, M. Merschmeyer, A. Meyer, S. Mukherjee, D. Noll, A. Novak, F. Nowotny, A. Pozdnyakov, Y. Rath, W. Redjeb, F. Rehm, H. Reithler, V. Sarkisovi, A. Schmidt, S. C. Schuler, A. Sharma, A. Stein, F. Torres Da Silva De Araujo, L. Vigilante, S. Wiedenbeck, S. Zaleski, C. Dziwok, G. Flügge, W. Haj Ahmad, T. Kress, A. Nowack, O. Pooth, A. Stahl, T. Ziemons, A. Zotz, H. Aarup Petersen, M. Aldaya Martin, J. Alimena, S. Amoroso, Y. An, S. Baxter, M. Bayatmakou, H. Becerril Gonzalez, O. Behnke, A. Belvedere, S. Bhattacharya, F. Blekman, K. Borras, D. Brunner, A. Campbell, A. Cardini, C. Cheng, F. Colombina, S. Consuegra Rodríguez, G. Correia Silva, M. De Silva, G. Eckerlin, D. Eckstein, L.I. Estevez Banos, O. Filatov, E. Gallo, A. Geiser, A. Giraldi, G. Greau, V. Guglielmi, M. Guthoff, A. Hinzmann, A. Jafari, L. Jeppe, N.Z. Jomhari, B. Kaech, M. Kasemann, H. Kaveh, C. Kleinwort, R. Kogler, M. Komm, D. Krücker, W. Lange, D. Leyva Pernia, K. Lipka, W. Lohmann, R. Mankel, I.-A. Melzer-Pellmann, M. Mendizabal Morentin, J. Metwally, A.B. Meyer, G. Milella, A. Mussgiller, A. Nürnberg, Y. Otarid, D. Pérez Adán, E. Ranken, A. Raspereza, B. Ribeiro Lopes, J. Rübenach, A. Saggio, M. Scham, V. Scheurer, S. Schnake, P. Schütze, C. Schwanenberger, M. Shchedrolosiev, R.E. Sosa Ricardo, L.P. Sreelatha Pramod, D. Stafford, F. Vazzoler, A. Ventura Barroso, R. Walsh, Q. Wang, Y. Wen, K. Wichmann, L. Wiens, C. Wissing, S. Wuchterl, Y. Yang, A. Zimermmane Castro Santos, A. Albrecht, S. Albrecht, M. Antonello, S. Bein, L. Benato, M. Bonanomi, P. Connor, M. Eich, K. El Morabit, Y. Fischer, A. Fröhlich, C. Garbers, E. Garutti, A. Grohsjean, M. Hajheidari, J. Haller, H.R. Jabusch, G. Kasieczka, P. Keicher, R. Klanner, W. Korcari, T. Kramer, V. Kutzner, F. Labe, J. Lange, A. Lobanov, C. Matthies, A. Mehta, L. Moureaux, M. Mrowietz, A. Nigamova, Y. Nissan, A. Paasch, K.J. Pena Rodriguez, T. Quadfasel, B. Raciti, M. Rieger, D. Savoiu, J. Schindler, P. Schleper, M. Schröder, J. Schwandt, M. Sommerhalder, H. Stadie, G. Steinbrück, A. Tews, M. Wolf, S. Brommer, M. Burkart, E. Butz, T. Chwalek, A. Dierlamm, A. Droll, N. Faltermann, M. Giffels, A. Gottmann, F. Hartmann, M. Horzela, U. Husemann, M. Klute, R. Koppenhöfer, M. Link, A. Lintuluoto, S. Maier, S. Mitra, M. Mormile, Th. Müller, M. Neukum, M. Oh, G. Quast, K. Rabbertz, I. Shvetsov, H.J. Simonis, N. Trevisani, R. Ulrich, J. van der Linden, R.F. Von Cube, M. Wassmer, S. Wieland, F. Wittig, R. Wolf, S. Wunsch, X. Zuo, G. Anagnostou, P. Assiouras, G. Daskalakis, A. Kyriakis, A. Papadopoulos, A. Stakia, D. Karasavvas, P. Kontaxakis, G. Melachroinos, A. Panagiotou, I. Papavergou, I. Paraskevas, N. Saoulidou, K. Theofilatos, E. Tziaferi, K. Vellidis, I. Zisopoulos, G. Bakas, T. Chatzistavrou, G. Karapostoli, K. Kousouris, I. Papakrivopoulos, E. Siamarkou, G. Tsipolitis, A. Zacharopoulou, K. Adamidis, I. Bestintzanos, I. Evangelou, C. Foudas, P. Gianneios, C. Kamtsikis, P. Katsoulis, P. Kokkas, P.G. Kosmoglou Kioseoglou, N. Manthos, I. Papadopoulos, J. Strologas, M. Csanád, K. Farkas, M.M.A. Gadallah, Á. Kadlecsik, P. Major, K. Mandal, G. Pásztor, A.J. Rádl, G.I. Veres, M. Bartók, C. Hajdu, D. Horvath, F. Sikler, V. Veszpremi, G. Bencze, S. Czellar, J. Karancsi, J. Molnar, Z. Szillasi, P. Raics, B. Ujvari, G. Zilizi, T. Csorgo, F. Nemes, T. Novak, J. Babbar, S. Bansal, S. B. Beri, V. Bhatnagar, G. Chaudhary, S. Chauhan, N. Dhingra, R. Gupta, A. Kaur, A. Kaur, H. Kaur, M. Kaur, S. Kumar, P. Kumari, M. Meena, K. Sandeep, T. Sheokand, J.B. Singh, A. Singla, A. Ahmed, A. Bhardwaj, A. Chhetri, B.C. Choudhary, A. Kumar, M. Naimuddin, K. Ranjan, S. Saumya, S. Baradia, S. Barman, S. Bhattacharya, D. Bhowmik, S. Dutta, S. Dutta, B. Gomber, P. Palit, G. Saha, B. Sahu, S. Sarkar, P.K. Behera, S.C. Behera, S. Chatterjee, P. Jana, P. Kalbhor, J.R. Komaragiri, D. Kumar, M. Mohammad Mobassir Ameen, L. Panwar, R. Pradhan, P.R. Pujahari, N.R. Saha, A. Sharma, A.K. Sikdar, S. Verma, T. Aziz, I. Das, S. Dugad, M. Kumar, G.B. Mohanty, P. Suryadevara, A. Bala, S. Banerjee, R. M. Chatterjee, M. Guchait, S. Karmakar, S. Kumar, G. Majumder, K. Mazumdar, S. Mukherjee, A. Thachayath, S. Bahinipati, A. K. Das, C. Kar, D. Maity, P. Mal, T. Mishra, V.K. Muraleedharan Nair Bindhu, K. Naskar, A. Nayak, P. Sadangi, P. Saha, S.K. Swain, S. Varghese, D. Vats, A. Alpana, S. Dube, B. Kansal, A. Laha, A. Rastogi, S. Sharma, H. Bakhshiansohi, E. Khazaie, M. Zeinali, S. Chenarani, S.M. Etesami, M. Khakzad, M. Mohammadi Najafabadi, M. Grunewald, M. Abbrescia, R. Aly, A. Colaleo, D. Creanza, B. D‘ Anzi, N. De Filippis, M. De Palma, A. Di Florio, W. Elmetenawee, L. Fiore, G. Iaselli, G. Maggi, M. Maggi, I. Margjeka, V. Mastrapasqua, S. My, S. Nuzzo, A. Pellecchia, A. Pompili, G. Pugliese, R. Radogna, G. Ramirez-Sanchez, D. Ramos, A. Ranieri, L. Silvestris, F.M. Simone, Ü. Sözbilir, A. Stamerra, R. Venditti, P. Verwilligen, A. Zaza, G. Abbiendi, C. Battilana, D. Bonacorsi, L. Borgonovi, R. Campanini, P. Capiluppi, A. Castro, F.R. Cavallo, G.M. Dallavalle, T. Diotalevi, F. Fabbri, A. Fanfani, D. Fasanella, P. Giacomelli, L. Giommi, C. Grandi, L. Guiducci, S. Lo Meo, L. Lunerti, S. Marcellini, G. Masetti, F.L. Navarria, A. Perrotta, F. Primavera, A.M. Rossi, T. Rovelli, G.P. Siroli, S. Costa, A. Di Mattia, R. Potenza, A. Tricomi, C. Tuve, G. Barbagli, G. Bardelli, B. Camaiani, A. Cassese, R. Ceccarelli, V. Ciulli, C. Civinini, R. D’Alessandro, E. Focardi, G. Latino, P. Lenzi, M. Lizzo, M. Meschini, S. Paoletti, A. Papanastassiou, G. Sguazzoni, L. Viliani, L. Benussi, S. Bianco, S. Meola, D. Piccolo, P. Chatagnon, F. Ferro, E. Robutti, S. Tosi, A. Benaglia, G. Boldrini, F. Brivio, F. Cetorelli, F. De Guio, M.E. Dinardo, P. Dini, S. Gennai, A. Ghezzi, P. Govoni, L. Guzzi, M.T. Lucchini, M. Malberti, S. Malvezzi, A. Massironi, D. Menasce, L. Moroni, M. Paganoni, D. Pedrini, B. S. Pinolini, S. Ragazzi, N. Redaelli, T. Tabarelli de Fatis, D. Zuolo, S. Buontempo, A. Cagnotta, F. Carnevali, N. Cavallo, A. De Iorio, F. Fabozzi, A.O.M. Iorio, L. Lista, P. Paolucci, B. Rossi, C. Sciacca, R. Ardino, P. Azzi, N. Bacchetta, D. Bisello, P. Bortignon, A. Bragagnolo, P. Checchia, T. Dorigo, F. Gasparini, U. Gasparini, A. Gozzelino, G. Grosso, M. Gulmini, L. Layer, E. Lusiani, M. Margoni, M. Migliorini, J. Pazzini, P. Ronchese, R. Rossin, F. Simonetto, G. Strong, M. Tosi, A. Triossi, S. Ventura, H. Yarar, M. Zanetti, P. Zotto, A. Zucchetta, G. Zumerle, S. Abu Zeid, C. Aimè, A. Braghieri, S. Calzaferri, D. Fiorina, P. Montagna, V. Re, C. Riccardi, P. Salvini, I. Vai, P. Vitulo, S. Ajmal, P. Asenov, G.M. Bilei, D. Ciangottini, L. Fanò, M. Magherini, G. Mantovani, V. Mariani, M. Menichelli, F. Moscatelli, A. Piccinelli, M. Presilla, A. Rossi, A. Santocchia, D. Spiga, T. Tedeschi, P. Azzurri, G. Bagliesi, R. Bhattacharya, L. Bianchini, T. Boccali, E. Bossini, D. Bruschini, R. Castaldi, M.A. Ciocci, M. Cipriani, V. D’Amante, R. Dell’Orso, S. Donato, A. Giassi, F. Ligabue, D. Matos Figueiredo, A. Messineo, M. Musich, F. Palla, S. Parolia, A. Rizzi, G. Rolandi, S. Roy Chowdhury, T. Sarkar, A. Scribano, P. Spagnolo, R. Tenchini, G. Tonelli, N. Turini, A. Venturi, P.G. Verdini, P. Barria, M. Campana, F. Cavallari, L. Cunqueiro Mendez, D. Del Re, E. Di Marco, M. Diemoz, F. Errico, E. Longo, P. Meridiani, J. Mijuskovic, G. Organtini, F. Pandolfi, R. Paramatti, C. Quaranta, S. Rahatlou, C. Rovelli, F. Santanastasio, L. Soffi, R. Tramontano, N. Amapane, R. Arcidiacono, S. Argiro, M. Arneodo, N. Bartosik, R. Bellan, A. Bellora, C. Biino, N. Cartiglia, M. Costa, R. Covarelli, N. Demaria, L. Finco, M. Grippo, B. Kiani, F. Legger, F. Luongo, C. Mariotti, S. Maselli, A. Mecca, E. Migliore, M. Monteno, R. Mulargia, M.M. Obertino, G. Ortona, L. Pacher, N. Pastrone, M. Pelliccioni, M. Ruspa, F. Siviero, V. Sola, A. Solano, D. Soldi, A. Staiano, C. Tarricone, M. Tornago, D. Trocino, G. Umoret, A. Vagnerini, E. Vlasov, S. Belforte, V. Candelise, M. Casarsa, F. Cossutti, K. De Leo, G. Della Ricca, S. Dogra, J. Hong, C. Huh, B. Kim, D.H. Kim, J. Kim, H. Lee, S.W. Lee, C.S. Moon, Y.D. Oh, S.I. Pak, M.S. Ryu, S. Sekmen, Y.C. Yang, G. Bak, P. Gwak, H. Kim, D.H. Moon, E. Asilar, D. Kim, T.J. Kim, J. A. Merlin, J. Park, S. Choi, S. Han, B. Hong, K. Lee, K.S. Lee, J. Park, S. K. Park, J. Yoo, J. Goh, H. S. Kim, Y. Kim, S. Lee, J. Almond, J. H. Bhyun, J. Choi, S. Jeon, W. Jun, J. Kim, J. S. Kim, S. Ko, H. Kwon, H. Lee, J. Lee, J. Lee, S. Lee, B.H. Oh, S.B. Oh, H. Seo, U. K. Yang, I. Yoon, W. Jang, D. Y. Kang, Y. Kang, S. Kim, B. Ko, J.S.H. Lee, Y. Lee, I.C. Park, Y. Roh, I.J. Watson, S. Yang, S. Ha, H.D. Yoo, M. Choi, M.R. Kim, H. Lee, Y. Lee, I. Yu, T. Beyrouthy, Y. Maghrbi, K. Dreimanis, A. Gaile, G. Pikurs, A. Potrebko, M. Seidel, V. Veckalns, N.R. Strautnieks, M. Ambrozas, A. Juodagalvis, A. Rinkevicius, G. Tamulaitis, N. Bin Norjoharuddeen, I. Yusuff, Z. Zolkapli, J.F. Benitez, A. Castaneda Hernandez, H. A. Encinas Acosta, L. G. Gallegos Maríñez, M. León Coello, J.A. Murillo Quijada, A. Sehrawat, L. Valencia Palomo, G. Ayala, H. Castilla-Valdez, E. De La Cruz-Burelo, I. Heredia-De La Cruz, R. Lopez-Fernandez, C. A. Mondragon Herrera, D.A. Perez Navarro, A. Sánchez Hernández, C. Oropeza Barrera, M. Ramírez García, I. Bautista, I. Pedraza, H.A. Salazar Ibarguen, C. Uribe Estrada, I. Bubanja, N. Raicevic, P.H. Butler, A. Ahmad, M. I. Asghar, A. Awais, M. I. M. Awan, H.R. Hoorani, W.A. Khan, V. Avati, L. Grzanka, M. Malawski, H. Bialkowska, M. Bluj, B. Boimska, M. Górski, M. Kazana, M. Szleper, P. Zalewski, K. Bunkowski, K. Doroba, A. Kalinowski, M. Konecki, J. Krolikowski, A. Muhammad, M. Araujo, D. Bastos, C. Beirão Da Cruz E Silva, A. Boletti, M. Bozzo, P. Faccioli, M. Gallinaro, J. Hollar, N. Leonardo, T. Niknejad, M. Pisano, J. Seixas, J. Varela, P. Adzic, P. Milenovic, M. Dordevic, J. Milosevic, V. Rekovic, M. Aguilar-Benitez, J. Alcaraz Maestre, M. Barrio Luna, Cristina F. Bedoya, M. Cepeda, M. Cerrada, N. Colino, B. De La Cruz, A. Delgado Peris, D. Fernández Del Val, J.P. Fernández Ramos, J. Flix, M.C. Fouz, O. Gonzalez Lopez, S. Goy Lopez, J.M. Hernandez, M.I. Josa, J. León Holgado, D. Moran, C. M. Morcillo Perez, Á. Navarro Tobar, C. Perez Dengra, A. Pérez-Calero Yzquierdo, J. Puerta Pelayo, I. Redondo, D.D. Redondo Ferrero, L. Romero, S. Sánchez Navas, L. Urda Gómez, J. Vazquez Escobar, C. Willmott, J.F. de Trocóniz, B. Alvarez Gonzalez, J. Cuevas, J. Fernandez Menendez, S. Folgueras, I. Gonzalez Caballero, J.R. González Fernández, E. Palencia Cortezon, C. Ramón Álvarez, V. Rodríguez Bouza, A. Soto Rodríguez, A. Trapote, C. Vico Villalba, P. Vischia, S. Bhowmik, S. Blanco Fernández, J.A. Brochero Cifuentes, I.J. Cabrillo, A. Calderon, J. Duarte Campderros, M. Fernandez, C. Fernandez Madrazo, G. Gomez, C. Lasaosa García, C. Martinez Rivero, P. Martinez Ruiz del Arbol, F. Matorras, P. Matorras Cuevas, E. Navarrete Ramos, J. Piedra Gomez, C. Prieels, L. Scodellaro, I. Vila, J.M. Vizan Garcia, M.K. Jayananda, B. Kailasapathy, D.U.J. Sonnadara, D.D.C. Wickramarathna, W.G.D. Dharmaratna, K. Liyanage, N. Perera, N. Wickramage, D. Abbaneo, C. Amendola, E. Auffray, G. Auzinger, J. Baechler, D. Barney, A. Bermúdez Martínez, M. Bianco, B. Bilin, A.A. Bin Anuar, A. Bocci, E. Brondolin, C. Caillol, T. Camporesi, G. Cerminara, N. Chernyavskaya, D. d’Enterria, A. Dabrowski, A. David, A. De Roeck, M.M. Defranchis, M. Deile, M. Dobson, F. Fallavollita, L. Forthomme, G. Franzoni, W. Funk, S. Giani, D. Gigi, K. Gill, F. Glege, L. Gouskos, M. Haranko, J. Hegeman, V. Innocente, T. James, P. Janot, J. Kieseler, S. Laurila, P. Lecoq, E. Leutgeb, C. Lourenço, B. Maier, L. Malgeri, M. Mannelli, A.C. Marini, F. Meijers, S. Mersi, E. Meschi, V. Milosevic, F. Moortgat, M. Mulders, S. Orfanelli, F. Pantaleo, M. Peruzzi, A. Petrilli, G. Petrucciani, A. Pfeiffer, M. Pierini, D. Piparo, H. Qu, D. Rabady, G. Reales Gutiérrez, M. Rovere, H. Sakulin, S. Scarfi, M. Selvaggi, A. Sharma, K. Shchelina, P. Silva, P. Sphicas, A.G. Stahl Leiton, A. Steen, S. Summers, D. Treille, P. Tropea, A. Tsirou, D. Walter, J. Wanczyk, K.A. Wozniak, P. Zehetner, P. Zejdl, W. D. Zeuner, T. Bevilacqua, L. Caminada, A. Ebrahimi, W. Erdmann, R. Horisberger, Q. Ingram, H.C. Kaestli, D. Kotlinski, C. Lange, M. Missiroli, L. Noehte, T. Rohe, T.K. Aarrestad, K. Androsov, M. Backhaus, A. Calandri, C. Cazzaniga, K. Datta, A. De Cosa, G. Dissertori, M. Dittmar, M. Donegà, F. Eble, M. Galli, K. Gedia, F. Glessgen, C. Grab, D. Hits, W. Lustermann, A.-M. Lyon, R.A. Manzoni, M. Marchegiani, L. Marchese, C. Martin Perez, A. Mascellani, F. Nessi-Tedaldi, F. Pauss, V. Perovic, S. Pigazzini, M.G. Ratti, M. Reichmann, C. Reissel, T. Reitenspiess, B. Ristic, F. Riti, D. Ruini, D.A. Sanz Becerra, R. Seidita, J. Steggemann, D. Valsecchi, R. Wallny, C. Amsler, P. Bärtschi, C. Botta, D. Brzhechko, M.F. Canelli, K. Cormier, A. De Wit, R. Del Burgo, J.K. Heikkilä, M. Huwiler, W. Jin, A. Jofrehei, B. Kilminster, S. Leontsinis, S.P. Liechti, A. Macchiolo, P. Meiring, V.M. Mikuni, U. Molinatti, I. Neutelings, A. Reimers, P. Robmann, S. Sanchez Cruz, K. Schweiger, M. Senger, Y. Takahashi, C. Adloff, C. M. Kuo, W. Lin, P.K. Rout, P.C. Tiwari, S.S. Yu, L. Ceard, Y. Chao, K.F. Chen, P. s. Chen, Z. g. Chen, W.-S. Hou, T. h. Hsu, Y. w. Kao, R. Khurana, G. Kole, Y.y. Li, R.-S. Lu, E. Paganis, A. Psallidas, X. f. Su, J. Thomas-Wilsker, H. y. Wu, E. Yazgan, C. Asawatangtrakuldee, N. Srimanobhas, V. Wachirapusitanand, D. Agyel, F. Boran, Z.S. Demiroglu, F. Dolek, I. Dumanoglu, E. Eskut, Y. Guler, E. Gurpinar Guler, C. Isik, O. Kara, A. Kayis Topaksu, U. Kiminsu, G. Onengut, K. Ozdemir, A. Polatoz, B. Tali, U.G. Tok, S. Turkcapar, E. Uslan, I.S. Zorbakir, K. Ocalan, M. Yalvac, B. Akgun, I.O. Atakisi, E. Gülmez, M. Kaya, O. Kaya, S. Tekten, A. Cakir, K. Cankocak, Y. Komurcu, S. Sen, O. Aydilek, S. Cerci, V. Epshteyn, B. Hacisahinoglu, I. Hos, B. Isildak, B. Kaynak, S. Ozkorucuklu, H. Sert, C. Simsek, D. Sunar Cerci, C. Zorbilmez, A. Boyaryntsev, B. Grynyov, L. Levchuk, D. Anthony, J.J. Brooke, A. Bundock, F. Bury, E. Clement, D. Cussans, H. Flacher, M. Glowacki, J. Goldstein, H.F. Heath, L. Kreczko, B. Krikler, S. Paramesvaran, S. Seif El Nasr-Storey, V.J. Smith, N. Stylianou, K. Walkingshaw Pass, R. White, A. H. Ball, K.W. Bell, A. Belyaev, C. Brew, R.M. Brown, D.J.A. Cockerill, C. Cooke, K. V. Ellis, K. Harder, S. Harper, M.-L. Holmberg, Sh. Jain, J. Linacre, K. Manolopoulos, D.M. Newbold, E. Olaiya, D. Petyt, T. Reis, G. Salvi, T. Schuh, C.H. Shepherd-Themistocleous, I.R. Tomalin, T. Williams, R. Bainbridge, P. Bloch, C.E. Brown, O. Buchmuller, V. Cacchio, C.A. Carrillo Montoya, G.S. Chahal, D. Colling, J. S. Dancu, P. Dauncey, G. Davies, J. Davies, M. Della Negra, S. Fayer, G. Fedi, G. Hall, M.H. Hassanshahi, A. Howard, G. Iles, M. Knight, J. Langford, L. Lyons, A.-M. Magnan, S. Malik, A. Martelli, M. Mieskolainen, J. Nash, M. Pesaresi, B.C. Radburn-Smith, A. Richards, A. Rose, C. Seez, R. Shukla, A. Tapper, K. Uchida, G.P. Uttley, L. H. Vage, T. Virdee, M. Vojinovic, N. Wardle, D. Winterbottom, K. Coldham, J.E. Cole, A. Khan, P. Kyberd, I.D. Reid, S. Abdullin, A. Brinkerhoff, B. Caraway, J. Dittmann, K. Hatakeyama, J. Hiltbrand, A.R. Kanuganti, B. McMaster, M. Saunders, S. Sawant, C. Sutantawibul, M. Toms, J. Wilson, R. Bartek, A. Dominguez, C. Huerta Escamilla, A.E. Simsek, R. Uniyal, A.M. Vargas Hernandez, R. Chudasama, S.I. Cooper, S.V. Gleyzer, C.U. Perez, P. Rumerio, E. Usai, C. West, R. Yi, A. Akpinar, A. Albert, D. Arcaro, C. Cosby, Z. Demiragli, C. Erice, E. Fontanesi, D. Gastler, J. Rohlf, K. Salyer, D. Sperka, D. Spitzbart, I. Suarez, A. Tsatsos, S. Yuan, G. Benelli, X. Coubez, D. Cutts, M. Hadley, U. Heintz, J.M. Hogan, T. Kwon, G. Landsberg, K.T. Lau, D. Li, J. Luo, S. Mondal, M. Narain, N. Pervan, S. Sagir, F. Simpson, W. Y. Wong, X. Yan, W. Zhang, S. Abbott, J. Bonilla, C. Brainerd, R. Breedon, M. Calderon De La Barca Sanchez, M. Chertok, M. Citron, J. Conway, P.T. Cox, R. Erbacher, G. Haza, F. Jensen, O. Kukral, G. Mocellin, M. Mulhearn, D. Pellett, B. Regnery, W. Wei, Y. Yao, F. Zhang, M. Bachtis, R. Cousins, A. Datta, J. Hauser, M. Ignatenko, M.A. Iqbal, T. Lam, E. Manca, W.A. Nash, D. Saltzberg, B. Stone, V. Valuev, R. Clare, M. Gordon, G. Hanson, W. Si, S. Wimpenny, J.G. Branson, S. Cittolin, S. Cooperstein, D. Diaz, J. Duarte, R. Gerosa, L. Giannini, J. Guiang, R. Kansal, V. Krutelyov, R. Lee, J. Letts, M. Masciovecchio, F. Mokhtar, M. Pieri, M. Quinnan, B.V. Sathia Narayanan, V. Sharma, M. Tadel, E. Vourliotis, F. Würthwein, Y. Xiang, A. Yagil, L. Brennan, C. Campagnari, G. Collura, A. Dorsett, J. Incandela, M. Kilpatrick, J. Kim, A.J. Li, P. Masterson, H. Mei, M. Oshiro, J. Richman, U. Sarica, R. Schmitz, F. Setti, J. Sheplock, D. Stuart, S. Wang, A. Bornheim, O. Cerri, A. Latorre, J.M. Lawhorn, J. Mao, H.B. Newman, T. Q. Nguyen, M. Spiropulu, J.R. Vlimant, C. Wang, S. Xie, R.Y. Zhu, J. Alison, S. An, M.B. Andrews, P. Bryant, V. Dutta, T. Ferguson, A. Harilal, C. Liu, T. Mudholkar, S. Murthy, M. Paulini, A. Roberts, A. Sanchez, W. Terrill, J.P. Cumalat, W.T. Ford, A. Hassani, G. Karathanasis, E. MacDonald, N. Manganelli, F. Marini, A. Perloff, C. Savard, N. Schonbeck, K. Stenson, K.A. Ulmer, S.R. Wagner, N. Zipper, J. Alexander, S. Bright-Thonney, X. Chen, D.J. Cranshaw, J. Fan, X. Fan, D. Gadkari, S. Hogan, J. Monroy, J.R. Patterson, J. Reichert, M. Reid, A. Ryd, J. Thom, P. Wittich, R. Zou, M. Albrow, M. Alyari, O. Amram, G. Apollinari, A. Apresyan, L.A.T. Bauerdick, D. Berry, J. Berryhill, P.C. Bhat, K. Burkett, J.N. Butler, A. Canepa, G.B. Cerati, H.W.K. Cheung, F. Chlebana, G. Cummings, J. Dickinson, I. Dutta, V.D. Elvira, Y. Feng, J. Freeman, A. Gandrakota, Z. Gecse, L. Gray, D. Green, S. Grünendahl, D. Guerrero, O. Gutsche, R.M. Harris, R. Heller, T.C. Herwig, J. Hirschauer, L. Horyn, B. Jayatilaka, S. Jindariani, M. Johnson, U. Joshi, T. Klijnsma, B. Klima, K.H.M. Kwok, S. Lammel, D. Lincoln, R. Lipton, T. Liu, C. Madrid, K. Maeshima, C. Mantilla, D. Mason, P. McBride, P. Merkel, S. Mrenna, S. Nahn, J. Ngadiuba, D. Noonan, V. Papadimitriou, N. Pastika, K. Pedro, C. Pena, F. Ravera, A. Reinsvold Hall, L. Ristori, E. Sexton-Kennedy, N. Smith, A. Soha, L. Spiegel, S. Stoynev, J. Strait, L. Taylor, S. Tkaczyk, N.V. Tran, L. Uplegger, E.W. Vaandering, I. Zoi, C. Aruta, P. Avery, D. Bourilkov, L. Cadamuro, P. Chang, V. Cherepanov, R. D. Field, E. Koenig, M. Kolosova, J. Konigsberg, A. Korytov, K. H. Lo, K. Matchev, N. Menendez, G. Mitselmakher, A. Muthirakalayil Madhu, N. Rawal, D. Rosenzweig, S. Rosenzweig, K. Shi, J. Wang, T. Adams, A. Al Kadhim, A. Askew, N. Bower, R. Habibullah, V. Hagopian, R. Hashmi, R.S. Kim, S. Kim, T. Kolberg, G. Martinez, H. Prosper, P. R. Prova, O. Viazlo, M. Wulansatiti, R. Yohay, J. Zhang, B. Alsufyani, M.M. Baarmand, S. Butalla, T. Elkafrawy, M. Hohlmann, R. Kumar Verma, M. Rahmani, F. Yumiceva, M.R. Adams, C. Bennett, R. Cavanaugh, S. Dittmer, R. Escobar Franco, O. Evdokimov, C.E. Gerber, D.J. Hofman, J.h. Lee, D. S. Lemos, A.H. Merrit, C. Mills, S. Nanda, G. Oh, B. Ozek, D. Pilipovic, T. Roy, S. Rudrabhatla, M.B. Tonjes, N. Varelas, X. Wang, Z. Ye, J. Yoo, M. Alhusseini, D. Blend, K. Dilsiz, L. Emediato, G. Karaman, O.K. Köseyan, J.-P. Merlo, A. Mestvirishvili, J. Nachtman, O. Neogi, H. Ogul, Y. Onel, A. Penzo, C. Snyder, E. Tiras, B. Blumenfeld, L. Corcodilos, J. Davis, A.V. Gritsan, L. Kang, S. Kyriacou, P. Maksimovic, M. Roguljic, J. Roskes, S. Sekhar, M. Swartz, T.Á. Vámi, A. Abreu, L.F. Alcerro Alcerro, J. Anguiano, P. Baringer, A. Bean, Z. Flowers, D. Grove, J. King, G. Krintiras, M. Lazarovits, C. Le Mahieu, C. Lindsey, J. Marquez, N. Minafra, M. Murray, M. Nickel, M. Pitt, S. Popescu, C. Rogan, C. Royon, R. Salvatico, S. Sanders, C. Smith, Q. Wang, G. Wilson, B. Allmond, A. Ivanov, K. Kaadze, A. Kalogeropoulos, D. Kim, Y. Maravin, K. Nam, J. Natoli, D. Roy, G. Sorrentino, F. Rebassoo, D. Wright, E. Adams, A. Baden, O. Baron, A. Belloni, A. Bethani, Y.M. Chen, S.C. Eno, N.J. Hadley, S. Jabeen, R.G. Kellogg, T. Koeth, Y. Lai, S. Lascio, A.C. Mignerey, S. Nabili, C. Palmer, C. Papageorgakis, M. M. Paranjpe, L. Wang, K. Wong, J. Bendavid, W. Busza, I.A. Cali, Y. Chen, M. D’Alfonso, J. Eysermans, C. Freer, G. Gomez-Ceballos, M. Goncharov, P. Harris, D. Hoang, D. Kovalskyi, J. Krupa, L. Lavezzo, Y.-J. Lee, K. Long, C. Mironov, C. Paus, D. Rankin, C. Roland, G. Roland, S. Rothman, Z. Shi, G.S.F. Stephans, J. Wang, Z. Wang, B. Wyslouch, T. J. Yang, B. Crossman, B.M. Joshi, C. Kapsiak, M. Krohn, D. Mahon, J. Mans, S. Pandey, M. Revering, R. Rusack, R. Saradhy, N. Schroeder, N. Strobbe, M.A. Wadud, L.M. Cremaldi, K. Bloom, M. Bryson, D.R. Claes, C. Fangmeier, F. Golf, J. Hossain, C. Joo, I. Kravchenko, I. Reed, J.E. Siado, G. R. Snow, W. Tabb, A. Wightman, F. Yan, D. Yu, A.G. Zecchinelli, G. Agarwal, H. Bandyopadhyay, L. Hay, I. Iashvili, A. Kharchilava, C. McLean, M. Morris, D. Nguyen, J. Pekkanen, S. Rappoccio, H. Rejeb Sfar, A. Williams, G. Alverson, E. Barberis, Y. Haddad, Y. Han, A. Krishna, J. Li, M. Lu, G. Madigan, B. Marzocchi, D.M. Morse, V. Nguyen, T. Orimoto, A. Parker, L. Skinnari, A. Tishelman-Charny, B. Wang, D. Wood, S. Bhattacharya, J. Bueghly, Z. Chen, K.A. Hahn, Y. Liu, Y. Miao, D.G. Monk, M.H. Schmitt, A. Taliercio, M. Velasco, R. Band, R. Bucci, S. Castells, M. Cremonesi, A. Das, R. Goldouzian, M. Hildreth, K.W. Ho, K. Hurtado Anampa, C. Jessop, K. Lannon, J. Lawrence, N. Loukas, L. Lutton, J. Mariano, N. Marinelli, I. Mcalister, T. McCauley, C. Mcgrady, K. Mohrman, C. Moore, Y. Musienko, H. Nelson, M. Osherson, R. Ruchti, A. Townsend, M. Wayne, H. Yockey, M. Zarucki, L. Zygala, A. Basnet, B. Bylsma, M. Carrigan, L.S. Durkin, C. Hill, M. Joyce, A. Lesauvage, M. Nunez Ornelas, K. Wei, B.L. Winer, B. R. Yates, F.M. Addesa, H. Bouchamaoui, P. Das, G. Dezoort, P. Elmer, A. Frankenthal, B. Greenberg, N. Haubrich, S. Higginbotham, G. Kopp, S. Kwan, D. Lange, A. Loeliger, D. Marlow, I. Ojalvo, J. Olsen, D. Stickland, C. Tully, S. Malik, A.S. Bakshi, V.E. Barnes, S. Chandra, R. Chawla, S. Das, A. Gu, L. Gutay, M. Jones, A.W. Jung, D. Kondratyev, A. M. Koshy, M. Liu, G. Negro, N. Neumeister, G. Paspalaki, S. Piperov, A. Purohit, J.F. Schulte, M. Stojanovic, J. Thieman, A. K. Virdi, F. Wang, W. Xie, J. Dolen, N. Parashar, A. Pathak, D. Acosta, A. Baty, T. Carnahan, S. Dildick, K.M. Ecklund, P.J. Fernández Manteca, S. Freed, P. Gardner, F.J.M. Geurts, A. Kumar, W. Li, O. Miguel Colin, B.P. Padley, R. Redjimi, J. Rotter, E. Yigitbasi, Y. Zhang, A. Bodek, P. de Barbaro, R. Demina, J.L. Dulemba, C. Fallon, A. Garcia-Bellido, O. Hindrichs, A. Khukhunaishvili, P. Parygin, E. Popova, R. Taus, G.P. Van Onsem, K. Goulianos, B. Chiarito, J.P. Chou, Y. Gershtein, E. Halkiadakis, A. Hart, M. Heindl, D. Jaroslawski, O. Karacheban, I. Laflotte, A. Lath, R. Montalvo, K. Nash, H. Routray, S. Salur, S. Schnetzer, S. Somalwar, R. Stone, S.A. Thayil, S. Thomas, J. Vora, H. Wang, H. Acharya, D. Ally, A.G. Delannoy, S. Fiorendi, T. Holmes, N. Karunarathna, L. Lee, E. Nibigira, S. Spanier, D. Aebi, M. Ahmad, O. Bouhali, M. Dalchenko, R. Eusebi, J. Gilmore, T. Huang, T. Kamon, H. Kim, S. Luo, S. Malhotra, R. Mueller, D. Overton, D. Rathjens, A. Safonov, N. Akchurin, J. Damgov, V. Hegde, A. Hussain, Y. Kazhykarim, K. Lamichhane, S.W. Lee, A. Mankel, T. Mengke, S. Muthumuni, T. Peltola, I. Volobouev, A. Whitbeck, E. Appelt, S. Greene, A. Gurrola, W. Johns, R. Kunnawalkam Elayavalli, A. Melo, F. Romeo, P. Sheldon, S. Tuo, J. Velkovska, J. Viinikainen, B. Cardwell, B. Cox, J. Hakala, R. Hirosky, A. Ledovskoy, A. Li, C. Neu, C.E. Perez Lara, P.E. Karchin, A. Aravind, S. Banerjee, K. Black, T. Bose, S. Dasu, I. De Bruyn, P. Everaerts, C. Galloni, H. He, M. Herndon, A. Herve, C.K. Koraka, A. Lanaro, R. Loveless, J. Madhusudanan Sreekala, A. Mallampalli, A. Mohammadi, S. Mondal, G. Parida, D. Pinna, A. Savin, V. Shang, V. Sharma, W.H. Smith, D. Teague, H.F. Tsoi, W. Vetens, A. Warden, S. Afanasiev, V. Andreev, Yu. Andreev, T. Aushev, M. Azarkin, A. Babaev, A. Belyaev, V. Blinov, E. Boos, V. Borshch, D. Budkouski, M. Chadeeva, V. Chekhovsky, R. Chistov, A. Dermenev, T. Dimova, D. Druzhkin, M. Dubinin, L. Dudko, A. Ershov, G. Gavrilov, V. Gavrilov, S. Gninenko, V. Golovtcov, N. Golubev, I. Golutvin, I. Gorbunov, A. Gribushin, Y. Ivanov, V. Kachanov, A. Kaminskiy, L. Kardapoltsev, V. Karjavine, A. Karneyeu, V. Kim, M. Kirakosyan, D. Kirpichnikov, M. Kirsanov, V. Klyukhin, O. Kodolova, D. Konstantinov, V. Korenkov, A. Kozyrev, N. Krasnikov, A. Lanev, P. Levchenko, N. Lychkovskaya, V. Makarenko, A. Malakhov, V. Matveev, V. Murzin, A. Nikitenko, S. Obraztsov, V. Oreshkin, V. Palichik, V. Perelygin, S. Petrushanko, S. Polikarpov, V. Popov, O. Radchenko, M. Savina, V. Savrin, D. Selivanova, V. Shalaev, S. Shmatov, S. Shulha, Y. Skovpen, S. Slabospitskii, V. Smirnov, A. Snigirev, D. Sosnov, V. Sulimov, E. Tcherniaev, A. Terkulov, O. Teryaev, I. Tlisova, A. Toropin, L. Uvarov, A. Uzunian, A. Vorobyev, N. Voytishin, B. S. Yuldashev, A. Zarubin, I. Zhizhin, A. Zhokin

**Affiliations:** 1 Yerevan Physics Institute, Yerevan, Armenia; 2 Institut für Hochenergiephysik, Vienna, Austria; 3 Universiteit Antwerpen, Antwerpen, Belgium; 4 Vrije Universiteit Brussel, Brussel, Belgium; 5 Université Libre de Bruxelles, Bruxelles, Belgium; 6 Ghent University, Ghent, Belgium; 7 Université Catholique de Louvain, Louvain-la-Neuve, Belgium; 8 Centro Brasileiro de Pesquisas Fisicas, Rio de Janeiro, Brazil; 9 Universidade do Estado do Rio de Janeiro, Rio de Janeiro, Brazil; 10 Universidade Estadual Paulista, Universidade Federal do ABC, São Paulo, Brazil; 11 Institute for Nuclear Research and Nuclear Energy, Bulgarian Academy of Sciences, Sofia, Bulgaria; 12 University of Sofia, Sofia, Bulgaria; 13 Instituto De Alta Investigación, Universidad de Tarapacá, Casilla 7 D, Arica, Chile; 14 Beihang University, Beijing, China; 15 Department of Physics, Tsinghua University, Beijing, China; 16 Institute of High Energy Physics, Beijing, China; 17 State Key Laboratory of Nuclear Physics and Technology, Peking University, Beijing, China; 18 Sun Yat-Sen University, Guangzhou, China; 19 University of Science and Technology of China, Hefei, China; 20 Institute of Modern Physics and Key Laboratory of Nuclear Physics and Ion-beam Application (MOE), Fudan University, Shanghai, China; 21 Zhejiang University, Hangzhou, Zhejiang China; 22 Universidad de Los Andes, Bogota, Colombia; 23 Universidad de Antioquia, Medellin, Colombia; 24 Faculty of Electrical Engineering, Mechanical Engineering and Naval Architecture, University of Split, Split, Croatia; 25 Faculty of Science, University of Split, Split, Croatia; 26 Institute Rudjer Boskovic, Zagreb, Croatia; 27 University of Cyprus, Nicosia, Cyprus; 28 Charles University, Prague, Czech Republic; 29 Escuela Politecnica Nacional, Quito, Ecuador; 30 Universidad San Francisco de Quito, Quito, Ecuador; 31 Academy of Scientific Research and Technology of the Arab Republic of Egypt, Egyptian Network of High Energy Physics, Cairo, Egypt; 32 Center for High Energy Physics (CHEP-FU), Fayoum University, El-Fayoum, Egypt; 33 National Institute of Chemical Physics and Biophysics, Tallinn, Estonia; 34 Department of Physics, University of Helsinki, Helsinki, Finland; 35 Helsinki Institute of Physics, Helsinki, Finland; 36 Lappeenranta-Lahti University of Technology, Lappeenranta, Finland; 37 IRFU, CEA, Université Paris-Saclay, Gif-sur-Yvette, France; 38 Laboratoire Leprince-Ringuet, CNRS/IN2P3, Ecole Polytechnique, Institut Polytechnique de Paris, Palaiseau, France; 39 Université de Strasbourg, CNRS, IPHC UMR 7178, Strasbourg, France; 40 Institut de Physique des 2 Infinis de Lyon (IP2I ), Villeurbanne, France; 41 Georgian Technical University, Tbilisi, Georgia; 42 I. Physikalisches Institut, RWTH Aachen University, Aachen, Germany; 43 III. Physikalisches Institut A, RWTH Aachen University, Aachen, Germany; 44 III. Physikalisches Institut B, RWTH Aachen University, Aachen, Germany; 45 Deutsches Elektronen-Synchrotron, Hamburg, Germany; 46 University of Hamburg, Hamburg, Germany; 47 Karlsruher Institut fuer Technologie, Karlsruhe, Germany; 48 Institute of Nuclear and Particle Physics (INPP), NCSR Demokritos, Aghia Paraskevi, Greece; 49 National and Kapodistrian University of Athens, Athens, Greece; 50 National Technical University of Athens, Athens, Greece; 51 University of Ioánnina, Ioannina, Greece; 52 MTA-ELTE Lendület CMS Particle and Nuclear Physics Group, Eötvös Loránd University, Budapest, Hungary; 53 Wigner Research Centre for Physics, Budapest, Hungary; 54 Institute of Nuclear Research ATOMKI, Debrecen, Hungary; 55 Institute of Physics, University of Debrecen, Debrecen, Hungary; 56 Karoly Robert Campus, MATE Institute of Technology, Gyongyos, Hungary; 57 Panjab University, Chandigarh, India; 58 University of Delhi, Delhi, India; 59 Saha Institute of Nuclear Physics, HBNI, Kolkata, India; 60 Indian Institute of Technology Madras, Madras, India; 61 Tata Institute of Fundamental Research-A, Mumbai, India; 62 Tata Institute of Fundamental Research-B, Mumbai, India; 63 National Institute of Science Education and Research, An OCC of Homi Bhabha National Institute, Bhubaneswar, Odisha India; 64 Indian Institute of Science Education and Research (IISER), Pune, India; 65 Isfahan University of Technology, Isfahan, Iran; 66 Institute for Research in Fundamental Sciences (IPM), Tehran, Iran; 67 University College Dublin, Dublin, Ireland; 68 INFN Sezione di Bari, Università di Bari, Politecnico di Bari, Bari, Italy; 69 INFN Sezione di Bologna, Università di Bologna, Bologna, Italy; 70 INFN Sezione di Catania, Università di Catania, Catania, Italy; 71 INFN Sezione di Firenze, Università di Firenze, Firenze, Italy; 72 INFN Laboratori Nazionali di Frascati, Frascati, Italy; 73 INFN Sezione di Genova, Università di Genova, Genoa, Italy; 74 INFN Sezione di Milano-Bicocca, Università di Milano-Bicocca, Milan, Italy; 75 INFN Sezione di Napoli, Università di Napoli ’Federico II’, Napoli, Italy; Università della Basilicata, Potenza, Italy;, Università G. Marconi, Rome, Italy; 76 INFN Sezione di Padova, Università di Padova, Padova, Italy; Università di Trento, Trento, Italy; 77 INFN Sezione di Pavia, Università di Pavia, Pavia, Italy; 78 INFN Sezione di Perugia, Università di Perugia, Perugia, Italy; 79 INFN Sezione di Pisa, Università di Pisa, Scuola Normale Superiore di Pisa, Pisa, Italy, Università di Siena, Siena, Italy; 80 INFN Sezione di Roma, Sapienza Università di Roma, Rome, Italy; 81 INFN Sezione di Torino, Università di Torino, Torino, Italy, Università del Piemonte Orientale, Novara, Italy; 82 INFN Sezione di Trieste, Università di Trieste, Trieste, Italy; 83 Kyungpook National University, Daegu, Korea; 84 Chonnam National University, Institute for Universe and Elementary Particles, Kwangju, Korea; 85 Hanyang University, Seoul, Korea; 86 Korea University, Seoul, Korea; 87 Kyung Hee University, Department of Physics, Seoul, Korea; 88 Sejong University, Seoul, Korea; 89 Seoul National University, Seoul, Korea; 90 University of Seoul, Seoul, Korea; 91 Yonsei University, Department of Physics, Seoul, Korea; 92 Sungkyunkwan University, Suwon, Korea; 93 College of Engineering and Technology, American University of the Middle East (AUM), Dasman, Kuwait; 94 Riga Technical University, Riga, Latvia; 95 University of Latvia (LU), Riga, Latvia; 96 Vilnius University, Vilnius, Lithuania; 97 National Centre for Particle Physics, Universiti Malaya, Kuala Lumpur, Malaysia; 98 Universidad de Sonora (UNISON), Hermosillo, Mexico; 99 Centro de Investigacion y de Estudios Avanzados del IPN, Mexico City, Mexico; 100 Universidad Iberoamericana, Mexico City, Mexico; 101 Benemerita Universidad Autonoma de Puebla, Puebla, Mexico; 102 University of Montenegro, Podgorica, Montenegro; 103 University of Canterbury, Christchurch, New Zealand; 104 National Centre for Physics, Quaid-I-Azam University, Islamabad, Pakistan; 105 AGH University of Krakow, Faculty of Computer Science, Electronics and Telecommunications, Kraków, Poland; 106 National Centre for Nuclear Research, Swierk, Poland; 107 Institute of Experimental Physics, Faculty of Physics, University of Warsaw, Warsaw, Poland; 108 Laboratório de Instrumentação e Física Experimental de Partículas, Lisbon, Portugal; 109 Faculty of Physics, University of Belgrade, Belgrade, Serbia; 110 VINCA Institute of Nuclear Sciences, University of Belgrade, Belgrade, Serbia; 111 Centro de Investigaciones Energéticas Medioambientales y Tecnológicas (CIEMAT), Madrid, Spain; 112 Universidad Autónoma de Madrid, Madrid, Spain; 113 Universidad de Oviedo, Instituto Universitario de Ciencias y Tecnologías Espaciales de Asturias (ICTEA), Oviedo, Spain; 114 Instituto de Física de Cantabria (IFCA), CSIC-Universidad de Cantabria, Santander, Spain; 115 University of Colombo, Colombo, Sri Lanka; 116 University of Ruhuna, Department of Physics, Matara, Sri Lanka; 117 CERN, European Organization for Nuclear Research, Geneva, Switzerland; 118 Paul Scherrer Institut, Villigen, Switzerland; 119 ETH Zurich-Institute for Particle Physics and Astrophysics (IPA), Zurich, Switzerland; 120 Universität Zürich, Zurich, Switzerland; 121 National Central University, Chung-Li, Taiwan; 122 National Taiwan University (NTU), Taipei, Taiwan; 123 Department of Physics, Faculty of Science, Chulalongkorn University, Bangkok, Thailand; 124 Physics Department, Science and Art Faculty, Çukurova University, Adana, Turkey; 125 Physics Department, Middle East Technical University, Ankara, Turkey; 126 Bogazici University, Istanbul, Turkey; 127 Istanbul Technical University, Istanbul, Turkey; 128 Istanbul University, Istanbul, Turkey; 129 Institute for Scintillation Materials of National Academy of Science of Ukraine, Kharkiv, Ukraine; 130 National Science Centre, Kharkiv Institute of Physics and Technology, Kharkiv, Ukraine; 131 University of Bristol, Bristol, UK; 132 Rutherford Appleton Laboratory, Didcot, UK; 133 Imperial College, London, UK; 134 Brunel University, Uxbridge, UK; 135 Baylor University, Waco, TX USA; 136 Catholic University of America, Washington, DC USA; 137 The University of Alabama, Tuscaloosa, AL USA; 138 Boston University, Boston, MA USA; 139 Brown University, Providence, RI USA; 140 University of California, Davis, Davis, CA USA; 141 University of California, Los Angeles, CA USA; 142 University of California, Riverside, Riverside, CA USA; 143 University of California, San Diego, La Jolla, CA USA; 144 Department of Physics, University of California, Santa Barbara, Santa Barbara, CA USA; 145 California Institute of Technology, Pasadena, CA USA; 146 Carnegie Mellon University, Pittsburgh, PA USA; 147 University of Colorado Boulder, Boulder, CO USA; 148 Cornell University, Ithaca, NY USA; 149 Fermi National Accelerator Laboratory, Batavia, IL USA; 150 University of Florida, Gainesville, FL USA; 151 Florida State University, Tallahassee, FL USA; 152 Florida Institute of Technology, Melbourne, FL USA; 153 University of Illinois Chicago, Chicago, Chicago, USA; 154 The University of Iowa, Iowa City, IA USA; 155 Johns Hopkins University, Baltimore, MD USA; 156 The University of Kansas, Lawrence, KS USA; 157 Kansas State University, Manhattan, KS USA; 158 Lawrence Livermore National Laboratory, Livermore, CA USA; 159 University of Maryland, College Park, MD USA; 160 Massachusetts Institute of Technology, Cambridge, MA USA; 161 University of Minnesota, Minneapolis, MN USA; 162 University of Mississippi, Oxford, MS USA; 163 University of Nebraska-Lincoln, Lincoln, NE USA; 164 State University of New York at Buffalo, Buffalo, NY USA; 165 Northeastern University, Boston, MA USA; 166 Northwestern University, Evanston, IL USA; 167 University of Notre Dame, Notre Dame, IN USA; 168 The Ohio State University, Columbus, OH USA; 169 Princeton University, Princeton, NJ USA; 170 University of Puerto Rico, Mayaguez, PR USA; 171 Purdue University, West Lafayette, IN USA; 172 Purdue University Northwest, Hammond, IN USA; 173 Rice University, Houston, TX USA; 174 University of Rochester, Rochester, NY USA; 175 The Rockefeller University, New York, NY USA; 176 Rutgers, The State University of New Jersey, Piscataway, NJ USA; 177 University of Tennessee, Knoxville, TN USA; 178 Texas A &M University, College Station, TX USA; 179 Texas Tech University, Lubbock, TX USA; 180 Vanderbilt University, Nashville, TN USA; 181 University of Virginia, Charlottesville, VA USA; 182 Wayne State University, Detroit, MI USA; 183 University of Wisconsin-Madison, Madison, WI USA; 184 Authors affiliated with an institute or an international laboratory covered by a cooperation agreement with CERN, Geneva, Switzerland; 185 Yerevan State University, Yerevan, Armenia; 186 TU Wien, Vienna, Austria; 187 Institute of Basic and Applied Sciences, Faculty of Engineering, Arab Academy for Science, Technology and Maritime Transport, Alexandria, Egypt; 188 Ghent University, Ghent, Belgium; 189 Universidade Estadual de Campinas, Campinas, Brazil; 190 Federal University of Rio Grande do Sul, Porto Alegre, Brazil; 191 UFMS, Nova Andradina, Brazil; 192 Nanjing Normal University, Nanjing, China; 193 Now at The University of Iowa, Iowa City, IA USA; 194 University of Chinese Academy of Sciences, Beijing, China; 195 University of Chinese Academy of Sciences, Beijing, China; 196 Université Libre de Bruxelles, Bruxelles, Belgium; 197 An institute or an international laboratory covered by a cooperation agreement with CERN, Geneva, Switzerland; 198 Helwan University, Cairo, Egypt; 199 Now at Zewail City of Science and Technology, Zewail, Egypt; 200 British University in Egypt, Cairo, Egypt; 201 Now at Ain Shams University, Cairo, Egypt; 202 Birla Institute of Technology, Mesra, Mesra, India; 203 Purdue University, West Lafayette, IN USA; 204 Université de Haute Alsace, Mulhouse, France; 205 Department of Physics, Tsinghua University, Beijing, China; 206 The University of the State of Amazonas, Manaus, Brazil; 207 Erzincan Binali Yildirim University, Erzincan, Turkey; 208 University of Hamburg, Hamburg, Germany; 209 III. Physikalisches Institut A, RWTH Aachen University, Aachen, Germany; 210 Isfahan University of Technology, Isfahan, Iran; 211 Bergische University Wuppertal (BUW), Wuppertal, Germany; 212 Brandenburg University of Technology, Cottbus, Germany; 213 Forschungszentrum Jülich, Juelich, Germany; 214 CERN, European Organization for Nuclear Research, Geneva, Switzerland; 215 Physics Department, Faculty of Science, Assiut University, Assiut, Egypt; 216 Wigner Research Centre for Physics, Budapest, Hungary; 217 Institute of Physics, University of Debrecen, Debrecen, Hungary; 218 Institute of Nuclear Research ATOMKI, Debrecen, Hungary; 219 Now at Universitatea Babes-Bolyai - Facultatea de Fizica, Cluj-Napoca, Romania; 220 Faculty of Informatics, University of Debrecen, Debrecen, Hungary; 221 Punjab Agricultural University, Ludhiana, India; 222 UPES-University of Petroleum and Energy Studies, Dehradun, India; 223 University of Visva-Bharati, Santiniketan, India; 224 University of Hyderabad, Hyderabad, India; 225 Indian Institute of Science (IISc), Bangalore, India; 226 IIT Bhubaneswar, Bhubaneswar, India; 227 Institute of Physics, Bhubaneswar, India; 228 Deutsches Elektronen-Synchrotron, Hamburg, Germany; 229 Department of Physics, Isfahan University of Technology, Isfahan, Iran; 230 Sharif University of Technology, Tehran, Iran; 231 Department of Physics, University of Science and Technology of Mazandaran, Behshahr, Iran; 232 Italian National Agency for New Technologies, Energy and Sustainable Economic Development, Bologna, Italy; 233 Centro Siciliano di Fisica Nucleare e di Struttura Della Materia, Catania, Italy; 234 Università degli Studi Guglielmo Marconi, Rome, Italy; 235 Scuola Superiore Meridionale, Università di Napoli ’Federico II’, Naples, Italy; 236 Fermi National Accelerator Laboratory, Batavia, IL USA; 237 Laboratori Nazionali di Legnaro dell’INFN, Legnaro, Italy; 238 Università di Napoli ’Federico II’, Naples, Italy; 239 Consiglio Nazionale delle Ricerche-Istituto Officina dei Materiali, Perugia, Italy; 240 Riga Technical University, Riga, Latvia; 241 Department of Applied Physics, Faculty of Science and Technology, Universiti Kebangsaan Malaysia, Bangi, Malaysia; 242 Consejo Nacional de Ciencia y Tecnología, Mexico City, Mexico; 243 Trincomalee Campus, Eastern University, Sri Lanka, Nilaveli, Sri Lanka; 244 INFN Sezione di Pavia, Università di Pavia, Pavia, Italy; 245 National and Kapodistrian University of Athens, Athens, Greece; 246 Ecole Polytechnique Fédérale Lausanne, Lausanne, Switzerland; 247 University of Vienna Faculty of Computer Science, Vienna, Austria; 248 Universität Zürich, Zurich, Switzerland; 249 Stefan Meyer Institute for Subatomic Physics, Vienna, Austria; 250 Laboratoire d’Annecy-le-Vieux de Physique des Particules, IN2P3-CNRS, Annecy-le-Vieux, France; 251 Near East University, Research Center of Experimental Health Science, Mersin, Turkey; 252 Konya Technical University, Konya, Turkey; 253 Izmir Bakircay University, Izmir, Turkey; 254 Adiyaman University, Adiyaman, Turkey; 255 Necmettin Erbakan University, Konya, Turkey; 256 Bozok Universitetesi Rektörlügü, Yozgat, Turkey; 257 Marmara University, Istanbul, Turkey; 258 Milli Savunma University, Istanbul, Turkey; 259 Kafkas University, Kars, Turkey; 260 Hacettepe University, Ankara, Turkey; 261 Faculty of Engineering, Istanbul University-Cerrahpasa, Istanbul, Turkey; 262 Yildiz Technical University, Istanbul, Turkey; 263 Vrije Universiteit Brussel, Brussel, Belgium; 264 School of Physics and Astronomy, University of Southampton, Southampton, UK; 265 University of Bristol, Bristol, UK; 266 IPPP Durham University, Durham, UK; 267 Monash University, Faculty of Science, Clayton, Australia; 268 Now at an institute or an international laboratory covered by a cooperation agreement with CERN, Geneva, Switzerland; 269 Università di Torino, Turin, Italy; 270 Bethel University, St. Paul, MN USA; 271 Karamanoğlu Mehmetbey University, Karaman, Turkey; 272 California Institute of Technology, Pasadena, CA USA; 273 United States Naval Academy, Annapolis, MD USA; 274 Bingol University, Bingol, Turkey; 275 Georgian Technical University, Tbilisi, Georgia; 276 Sinop University, Sinop, Turkey; 277 Erciyes University, Kayseri, Turkey; 278 Horia Hulubei National Institute of Physics and Nuclear Engineering (IFIN-HH), Bucharest, Romania; 279 Texas A &M University at Qatar, Doha, Qatar; 280 Kyungpook National University, Daegu, Korea; 281 Another institute or international laboratory covered by a cooperation agreement with CERN, Geneva, Switzerland; 282 Universiteit Antwerpen, Antwerpen, Belgium; 283 Yerevan Physics Institute, Yerevan, Armenia; 284 Northeastern University, Boston, MA USA; 285 Imperial College, London, UK; 286 Institute of Nuclear Physics of the Uzbekistan Academy of Sciences, Tashkent, Uzbekistan; 287 CERN, Geneva, Switzerland

## Abstract

The measurement of Z boson production is presented as a method to determine the integrated luminosity of CMS data sets. The analysis uses proton–proton collision data, recorded by the CMS experiment at the CERN LHC in 2017 at a center-of-mass energy of 13$\,\text {Te\hspace{-.08em}V}$. Events with Z bosons decaying into a pair of muons are selected. The total number of Z bosons produced in a fiducial volume is determined, together with the identification efficiencies and correlations from the same data set, in small intervals of 20$\,\text {pb}^{-1}$ of integrated luminosity, thus facilitating the efficiency and rate measurement as a function of time and instantaneous luminosity. Using the ratio of the efficiency-corrected numbers of Z bosons, the precisely measured integrated luminosity of one data set is used to determine the luminosity of another. For the first time, a full quantitative uncertainty analysis of the use of Z  bosons for the integrated luminosity measurement is performed. The uncertainty in the extrapolation between two data sets, recorded in 2017 at low and high instantaneous luminosity, is less than 0.5%. We show that the Z boson rate measurement constitutes a precise method, complementary to traditional methods, with the potential to improve the measurement of the integrated luminosity.

## Introduction

In the CERN LHC, during the Run 2 data-taking period in 2015–2018, about 300 million events with Z bosons decaying into pairs of muons were recorded by the CMS experiment. Precision cross section measurements were performed [1–6] that provide (i) important tests of theoretical calculations [7–9]; (ii) input to fits of the parton distribution functions (PDFs) of the proton [10–13]; and (iii) constraints on backgrounds to searches for new physics [14].

Events with a Z boson decaying into a pair of muons have a remarkably clean experimental signature and a large cross section that facilitates high-precision measurements. Samples of Z bosons are also used as standard tools for detector calibrations and efficiency studies. The precisely known Z boson mass and width [15] are used to calibrate energy scales and momenta and to determine the detector resolution [16, 17]. Efficiencies for lepton triggering, reconstruction, and identification are determined using the “tag-and-probe” method [1, 16–18].

The large Drell–Yan (DY) cross section for the production of Z  bosons, and the possibility of simultaneously determining both the yield and the detection efficiency in situ, i.e., from the same event sample, make the process useful for precision measurements of the integrated luminosity. This was discussed before the start of the LHC [19]. During LHC operation, measurements of the Z boson rate already proved to be a useful and independent method for the LHC machine operators and experiments to monitor the relative instantaneous luminosity delivered to the ATLAS and CMS experiments [20]. The use of Z boson production as a measure of relative luminosities was also explored by the ATLAS experiment [21].

Both muons from the Z boson decay are detectable within the fiducial volume of the CMS detector in about one third of the Z  boson events. The fiducial Z boson cross section in proton–proton ($\text {pp}$) collisions at 13$\,\text {Te\hspace{-.08em}V}$ has been measured to be [3]

1 $$\begin{aligned} \sigma ^{\textrm{Z}} \mathcal {B} ({\textrm{Z}}\rightarrow {\mu ^{+}} {\mu ^{-}}) = 694 \pm 6 \,\text {(syst)} \pm 17 \,\text {(lumi)} \text {\,pb}. \end{aligned}$$

Theoretical predictions are available up to next-to-next-to-next-to-leading order (N^3^LO) [9] in quantum chromodynamics (QCD). Electroweak corrections, including mixed QCD-electroweak corrections, are also available [7, 22, 23]. The current uncertainty in the prediction of the fiducial cross section is about 3%, and mainly originates from limited knowledge of proton PDFs and higher-order corrections [8]. Within this uncertainty, the integrated luminosity can be directly determined from the measured number of Z bosons corrected for efficiencies.

In practice, precision luminosity calibrations at the LHC are obtained from van der Meer (vdM) scan data [21, 24–29], which are more precise than the theory predictions for the Z boson cross section. In vdM scans, which are performed at low instantaneous luminosity with zero crossing angle between the two beams, the two beams are separated in two orthogonal directions transverse to the parallel beam axes. In each scan step, for a given beam separation, the event rate measured in the luminosity detectors is recorded to determine the beam overlap area. Together with the beam currents and the measured head-on collision rate, a luminosity calibration constant, referred to as the visible cross section, is determined. A full vdM scan campaign takes about six hours per experiment and is usually performed once per year, with specifically configured beams to maximize the accuracy and precision of the measurement. A detailed description of vdM scans is reported in Ref. [29].

The most precise integrated luminosity measurement in CMS to date, achieved for the 2016 data-taking period, has a total uncertainty of 1.2% [29]. Roughly half of the total uncertainty is due to the luminosity integration over the full year of data taking. This uncertainty, in turn, is composed of the uncertainty in the extrapolation of the visible cross section obtained in the vdM scan to standard data-taking conditions at high instantaneous luminosity, and the uncertainty in the integration of the instantaneous luminosity over time, obtained from comparisons between different luminosity measurements. In the 2017 data, presented in this paper, the average number of collisions per bunch crossing, usually referred to as pileup, was 32 [30]. In Run 2, peak instantaneous luminosities as high as 20$\,\text {nb}^{-1}\,\text {s}^{-1}$ were reached, corresponding to a pileup of more than 50. For the high-luminosity LHC (HL-LHC), a pileup of up to 200 is expected [31] likely leading to an increase in uncertainty with the conventional methods due to the larger extrapolation.

In this paper, we explore an approach originally proposed in Ref. [32]. The measurement of the Z boson rate is used as an alternative method for the extrapolation and integration of the luminosity calibration. The Z boson counting complements conventional luminosity measurements obtained from the CMS luminosity systems, which are taken as reference luminosity. The fiducial Z boson production cross section is defined as $\sigma ^{\textrm{Z}}_\text {fid} =N^{{\textrm{Z}}}/\mathcal {L}$, where $N^{{\textrm{Z}}}$ stands for the efficiency-corrected number of reconstructed Z boson events and $\mathcal {L}$ for the integrated luminosity. Since $\sigma ^{\textrm{Z}}_\text {fid}$ is identical for all data sets of the same center-of-mass energy, the ratio of $N^{{\textrm{Z}}}$ for two data sets can be used to transfer the luminosity calibration from one data set to another, without input from theoretical predictions or precise knowledge of $\sigma ^{\textrm{Z}}_\text {fid}$. For the first time, a full quantitative uncertainty analysis of the use of Z bosons for the integrated luminosity measurement is performed.

We choose two independent data sets of Z boson events, both recorded by the CMS experiment in 2017: a data set with a bunch luminosity corresponding to about three $\text {pp}$ collisions per bunch crossing, referred to in the following as “$\textrm{lowPU}$ ”; and the bulk of CMS $\text {pp}$ collision data recorded in 2017 with a typical pileup of 32, denoted as “$\textrm{highPU}$ ”. The luminosity of the $\textrm{lowPU}$ data is used to determine that of the $\textrm{highPU}$ data via the relation

2 $$\begin{aligned} \mathcal {L} _\textrm{highPU} = \frac{N^{{\textrm{Z}}} _\textrm{highPU}}{N^{{\textrm{Z}}} _\textrm{lowPU}} \mathcal {L} _\textrm{lowPU}. \end{aligned}$$

For both sets of data, the individual trigger and selection efficiencies are determined in situ, in intervals of 20$\,\text {pb}^{-1}$, thus enhancing the sensitivity to possible variations due to changes in beam conditions or detector response as a function of time. Using the integrated luminosity for the $\textrm{lowPU}$ data, which has an uncertainty of 1.7% [33], the integrated luminosity $\mathcal {L} _\textrm{highPU}$ and its uncertainty are determined from Eq. (2), and compared with the result from the conventional integrated luminosity measurement. Due to the cleaner signature, better resolution, and smaller backgrounds, the analysis of Z boson decays into muons is more accurate than electrons. In this paper, only the decays of Z bosons into muons are used.

The paper is structured as follows. After a brief outline of the CMS detector in Sect. 2, the analysis of the Z boson event sample is described in Sect. 3. The reconstructed number of Z bosons and the trigger and selection efficiencies are extracted from fits to the data. Acceptance corrections and correlations between the efficiencies for the muon track components and among the two muon tracks are studied as a function of pileup. Subsequently, in Sect. 4, the luminosity information obtained from Z boson counting is compared with the results from conventional luminosity measurements. In Sect. 5, the benefits and advantages of Z boson counting for luminosity measurements are discussed. The paper concludes with a summary in Sect. 6.

## The CMS detector

The central feature of the CMS apparatus is a superconducting solenoid of 6$\text {\,m}$ internal diameter, providing a magnetic field of 3.8$\text {\,T}$. Within the magnet volume are a silicon pixel and strip tracker, a lead tungstate crystal electromagnetic calorimeter (ECAL), and a brass and scintillator hadron calorimeter, each composed of a barrel and two endcap sections. Forward calorimeters extend the pseudorapidity ($\eta$) coverage provided by the barrel and endcap detectors. The muon system consists of gas-ionization detectors embedded in the steel flux-return yoke outside the solenoid. A more detailed description of the CMS detector, together with a definition of the coordinate system used and the relevant kinematic variables, is reported in Ref. [34].

The silicon tracker measures charged particles in the pseudorapidity range $|\eta | <3.0$ [35, 36]. An iterative approach is used to build tracker tracks, executing a sequence of tracking algorithms, each with slightly distinct logic [17]. Muons are measured in the range $|\eta | <2.4$, with detection planes made using three technologies: drift tubes, cathode strip chambers, and resistive plate chambers. Matching muons to tracks measured in the silicon tracker results in a relative transverse momentum ($p_{\textrm{T}}$) resolution of 1% in the barrel and 3% in the endcaps, for muons with $p_{\textrm{T}}$ of about 100$\,\text {Ge\hspace{-.08em}V}$  [17]. The particle-flow (PF) algorithm [37] reconstructs and identifies each individual particle in an event, combining information from the various CMS detector components. Jets are clustered using the anti-$k_{\textrm{T}}$ jet finding algorithm [38, 39] with the tracks assigned to candidate vertices as inputs, and the associated missing transverse momentum $p_{\textrm{T}} ^\text {miss}$, taken as the negative vector $p_{\textrm{T}}$ sum of those jets [40]. The primary vertex (PV) is taken to be the vertex with the largest $\sum p_{\textrm{T}} ^2$ of its associated tracks, as described in Section 9.4 of Ref. [41].

Events of interest are selected using a two-tiered trigger system. The first level (L1), comprised of custom hardware processors, uses information from the calorimeters and muon detectors to select events at a rate of around 100$\text {\,kHz}$ within a fixed latency of 4 $\upmu$ s [42]. The second level, known as the high-level trigger (HLT), consists of a farm of processors running a version of the full event reconstruction software optimized for fast processing, and reduces the event rate to around 1$\text {\,kHz}$ before data storage [43].

During LHC Run 2, the main CMS luminosity subdetectors (luminometers) were the silicon pixel detector, the hadron forward calorimeter (HF), the pixel luminosity telescope (PLT) [44], and the fast beam conditions monitor (BCM1F) [45]. A separate data acquisition system is used to collect and store HF, PLT, and BCM1F data, as well as LHC beam-related data. A more detailed description of the CMS luminosity system is reported in Ref. [29]. For all comparisons in this paper, the reference integrated luminosity is obtained with the CMS luminometers, calibrated as described in Ref. [33] and using offline-calibrated corrections for the afterglow effects in the HF luminosity measurement.

The analysis described in this paper is largely independent of Monte Carlo (MC) simulations. However, MC simulations are used for two purposes: to determine the expected DY invariant mass distribution of the signal measured in the CMS detector; and to study possible biases in the pileup-dependent measurement of the muon track-finding efficiencies. Simulated event samples of the DY process, ${\textrm{Z}}/\gamma ^*\rightarrow \ell \ell$, are produced at leading order using the MadGraph 5_amc@nlo (v2.6.5) [46] generator, interfaced with pythia (v8.240) [47] for the parton shower simulation. The parameters describing the modeling of the parton shower and underlying event are based on the CP5 tune [48]. The generated MC events are passed through a full simulation of the detector using Geant4  [49].

## The Z boson candidate selection and efficiency determination

The events were recorded using a single-muon trigger (HLT muon) that requires at least one muon candidate with $p_{\textrm{T}} >24\,\text {Ge\hspace{-.08em}V}$ and loose isolation criteria [50]. The $\textrm{lowPU}$ data were recorded using different, looser trigger configurations than those used for the $\textrm{highPU}$ data. To obtain identical trigger configurations for the two data sets, the trigger decision in the $\textrm{lowPU}$ was recalculated from raw data using the trigger configuration of the $\textrm{highPU}$ data.

Based on the offline reconstruction, selected muon candidates consist of an “outer” standalone track in the muon system, matched to an “inner” track reconstructed in the silicon tracker [35]. The outer track is required to have signals in at least two muon detector planes. The inner track must have at least one valid hit in the silicon pixel detector and hits in more than five strip tracker layers. The matching is done by comparing parameters of the two tracks propagated onto a common surface. A combined Kalman filter fit [51] is performed in which the information from the inner and outer tracks is used to obtain a “global” muon track. For global muons, the inner and outer tracks are required to have $p_{\textrm{T}} >20\,\text {Ge\hspace{-.08em}V}$, lie within $|\eta | <2.4$, and to be matched within $\Delta R= \sqrt{\smash [b]{(\Delta \eta )^2 + (\Delta \phi )^2}} < 0.3$. Quality criteria on the global muon track fit are imposed, and it is required that the muon candidate is also reconstructed with the PF algorithm [37]. No requirements are imposed on the impact parameters of the muon track. Isolation criteria are omitted to maintain efficiency also at high pileup. For muons with $p_{\textrm{T}} <200\,\text {Ge\hspace{-.08em}V}$, i.e., about 99% of identified muon candidates, the track parameters are taken from the inner track. In other cases, the track parameters are determined by combining information from the inner and outer tracks. For all muon tracks, $p_{\textrm{T}} >25\,\text {Ge\hspace{-.08em}V}$ is required to ensure that the trigger efficiency reaches a plateau.

A Z boson candidate is identified as a pair of opposite-charge muons with an invariant mass of $60< m_{\upmu \upmu } < 120\,\text {Ge\hspace{-.08em}V}$. At least one of the two muon candidates is required to be matched with an HLT muon within $\Delta R< 0.1$. To obtain the actual number of produced Z bosons, the number of reconstructed and selected Z  boson candidates, the trigger efficiency, the muon-identification efficiency, as well as the background arising from nonresonant production, are determined from dedicated fits to the data, as explained in the following.

### Trigger efficiency and signal extraction

The trigger efficiency and the number of Z boson candidates are determined from fits to the invariant dimuon mass distributions of mutually exclusive sets of events with exactly one ($N_{1}$) or exactly two ($N_{2}$) selected muons matched to an HLT muon. The observables $N_{1}$ and $N_{2}$ follow the relations

3 $$\begin{aligned} \begin{aligned} N_{1}&= 2 \epsilon ^{\upmu }_\textrm{HLT} \big (1 - C_{\textrm{HLT}} \epsilon ^{\upmu }_\textrm{HLT} \big ) \epsilon ^{{\textrm{Z}}}_\textrm{ID} N^{{\textrm{Z}}} + N_{1}^{\text {bkg}}, \\ N_{2}&= C_{\textrm{HLT}} \big (\epsilon ^{\upmu }_\textrm{HLT} \big )^2 \epsilon ^{{\textrm{Z}}}_\textrm{ID} N^{{\textrm{Z}}} + N_{2}^{\text {bkg}}. \end{aligned} \end{aligned}$$

Here, the quantity $\epsilon ^{\upmu }_\textrm{HLT}$ refers to the HLT muon trigger efficiency. The correction factor $C_{\textrm{HLT}}$ accounts for the correlation between the HLT efficiencies of the two muons. A value of $C_{\textrm{HLT}} >1$ indicates a positive correlation between the two muons, i.e., an increased probability for the second muon to pass the HLT if the first muon passes it. The determination of $C_{\textrm{HLT}}$ is presented in Sect. 3.2. The terms $N_{1}^{\text {bkg}}$ and $N_{2}^{\text {bkg}}$ describe the contributions from nonresonant backgrounds. The reconstruction efficiency $\epsilon ^{{\textrm{Z}}}_\textrm{ID}$ is separately determined from the data, as described in Sect. 3.3.

**Fig. 1:**
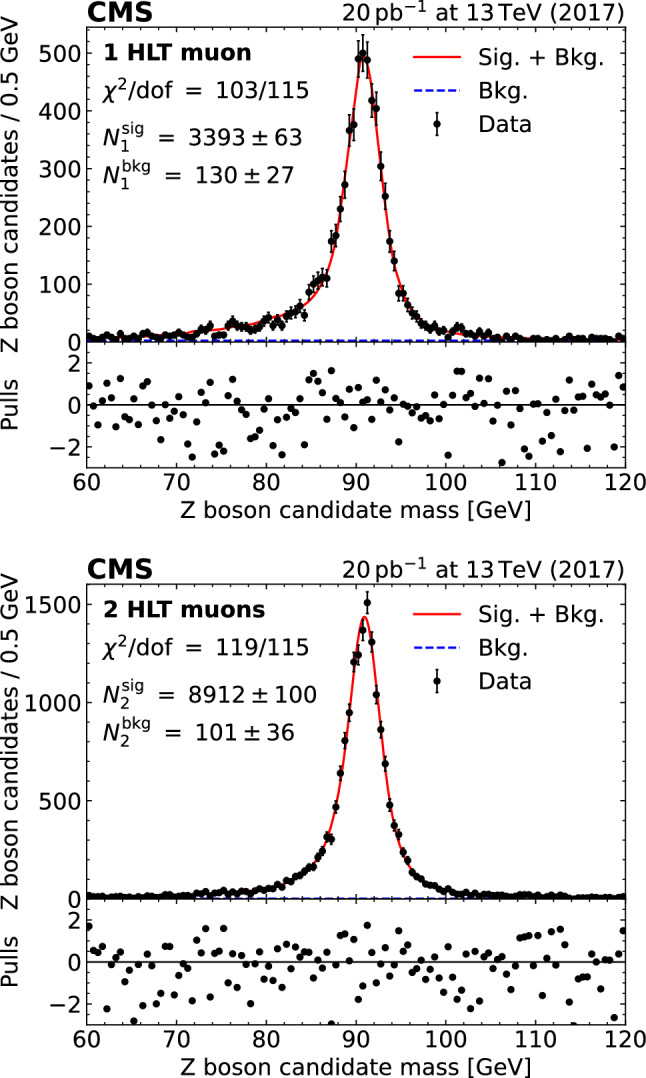
The upper panels show the reconstructed invariant mass distributions of Z boson candidates in a 20$\,\text {pb}^{-1}$ sample of data for events where one (upper) or two (lower) muons pass the single-muon trigger selection. The blue curve shows the fitted background contribution and the red curve illustrates the modeled signal-plus-background contribution. The error bars indicate the statistical uncertainties. The numbers of signal and background candidates are given by $N_i^{\text {sig}} =N_i- N_i^{\text {bkg}}$ and $N_i^{\text {bkg}}$, respectively. Also indicated are the $\chi ^2$ values per degree of freedom (dof). The lower panels contain the pulls of the distributions, defined as the difference between the data and the fit model in each bin, divided by the statistical uncertainty estimated from the expected number of entries given by the model

A fit is performed to two histograms binned in $m_{\upmu \upmu }$ for Z  candidates contributing to $N_{1}$ and $N_{2}$ in which $\epsilon ^{\upmu }_\textrm{HLT}$ and $N^{{\textrm{Z}}}$ are two free parameters. In the fit, the signal is modeled by a histogram template generated from simulated ${\textrm{Z}}\rightarrow \upmu \upmu$ events, convolved with a Gaussian function to take into account muon momentum scale and resolution differences between data and simulation. A falling exponential function is used to describe the nonresonant background. In Fig. 1, examples of two distributions and the results of the fits are presented. The sample shown here corresponds to an integrated luminosity of 20$\,\text {pb}^{-1}$, yielding about 12 000 Z boson candidates.

### Muon trigger correlation

The correlation between the trigger efficiencies of the two HLT muons is described by the correction factor $C_{\textrm{HLT}}$, as introduced in Eq. (3). The dependence of $C_{\textrm{HLT}}$ on the pileup is of particular interest in this analysis because it does not cancel in the ratio in Eq. (2), and thus constitutes an important source of systematic uncertainty. The correlation was investigated in simulation, and it is largely understood to originate from isolation requirements in the trigger selection.

We determine $C_{\textrm{HLT}}$ from an MC simulation sample of ${\textrm{Z}}\rightarrow \upmu \upmu$ events. As a proxy to the amount of pileup in a given event, we use the number of reconstructed PVs, $N_{\textrm{PV}}$, an observable that is directly accessible event-by-event in both data and simulation. At fixed pileup, the distribution of $N_{\textrm{PV}}$ approximately follows a Poisson distribution with a mean at about 80% of the true pileup, as determined from DY simulation.

In the simulation, $C_{\textrm{HLT}}$ is obtained directly, by rearranging Eq. (3), as

4 $$\begin{aligned} C_{\textrm{HLT}} = \frac{4 N^{{\textrm{Z}}} \epsilon ^{{\textrm{Z}}}_\textrm{ID} N_{2}^{\text {sig}}}{\left( N_{1}^{\text {sig}} + 2 N_{2}^{\text {sig}} \right) ^2}, \end{aligned}$$

where $N_{1}^{\text {sig}}$ and $N_{2}^{\text {sig}}$ are the number of signal events, corresponding to $N_{1}-N_{1}^{\text {bkg}}$ and $N_{2}-N_{2}^{\text {bkg}}$ in the data.

We use data to validate the result for $C_{\textrm{HLT}}$ obtained in the simulation. To this end, events are analyzed that are triggered independently of the muon trigger, namely by using the trigger condition $p_{\textrm{T}} ^\text {miss} >120\,\text {Ge\hspace{-.08em}V}$ in which the contribution from muons is not included. This $p_{\textrm{T}} ^\text {miss}$ trigger also records Z boson candidates for which the number of HLT muons is zero, and, thus, an additional relation for the number of reconstructed Z boson candidates with no HLT muons, denoted as $N_{0}$, is obtained,

5 $$\begin{aligned} N_{0} = \big (1-2\epsilon ^{\upmu }_\textrm{HLT} + C_{\textrm{HLT}} (\epsilon ^{\upmu }_\textrm{HLT})^2 \big ) \epsilon ^{{\textrm{Z}}}_\textrm{ID} N^{{\textrm{Z}}} + N_{0}^{\text {bkg}}. \end{aligned}$$

Together with Eq. (3), we obtain three equations for $N_{0}$, $N_{1}$, and $N_{2}$ with three unknowns, $\epsilon ^{\upmu }_\textrm{HLT}$, $C_{\textrm{HLT}}$, and $\epsilon ^{{\textrm{Z}}}_\textrm{ID} N^{{\textrm{Z}}}$. The correction factor $C_{\textrm{HLT}}$ can thus be determined from the number of signal events in the three categories, each obtained from a fit. The fits are performed separately in six bins of $N_{\textrm{PV}}$ where the number of bins and their boundaries are chosen such that the number of events per bin are similar.

**Fig. 2:**
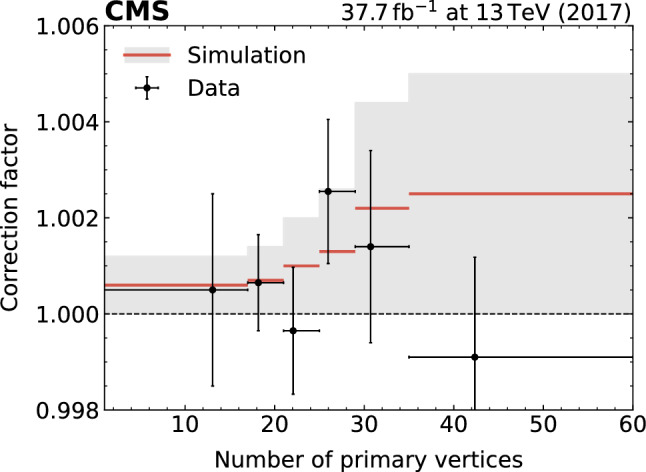
Correction factor $C_{\textrm{HLT}}$ for the correlation between the measured muon trigger efficiencies of the two muons as a function of the number of reconstructed primary vertices, $N_{\textrm{PV}}$, in the simulation (lines) and the data (points). The data points are drawn at the mean value of $N_{\textrm{PV}}$ in each bin of the measurement. The horizontal error bars on the points show the bin width, and the vertical error bars show the statistical uncertainty. The gray band indicates the $\pm 100\%$ uncertainty in the correction factor

The result is presented in Fig. 2. The red lines indicate the expectation from the simulation in which $C_{\textrm{HLT}}$ is at the level of 0.1–0.2% above unity for $N_{\textrm{PV}} \sim 30$. Within the limited statistical precision of the data, good agreement of the simulation with the data is observed. We assign a systematic uncertainty of 100% of the correction, which is represented by the gray band in the figure.

### Muon identification and reconstruction efficiency

The efficiency to reconstruct a Z boson, $\epsilon ^{{\textrm{Z}}}_\textrm{ID}$, depends on the muon identification and reconstruction efficiency $\epsilon ^{\upmu }_\textrm{ID}$ for each of the two muons. In the simulation, the pileup-dependent correlation between the two identified muons is of the order of 0.01%, and thus $\epsilon ^{{\textrm{Z}}}_\textrm{ID} = C_{\textrm{ID}} \big ( \epsilon ^{\upmu }_\textrm{ID} \big )^2$. The value for $C_{\textrm{ID}} \approx 1.0001$ is taken from simulation and applied as a function of $N_{\textrm{PV}}$. The muon efficiency $\epsilon ^{\upmu }_\textrm{ID}$ is defined independently of the HLT muon efficiency, such that the total number of produced Z bosons is obtained from Eq. (3).

To determine $\epsilon ^{\upmu }_\textrm{ID}$, the following factorization ansatz is used:

6 $$ \begin{aligned} \epsilon ^{\upmu }_\textrm{ID} = \epsilon ^{\upmu }_\mathrm {ID|Glo} \, \epsilon ^{\upmu }_\mathrm {Glo|Sta} \, \epsilon ^{\upmu }_\mathrm {Sta|Trk} \, \frac{1}{c_{\mathrm {T \& P}}}, \end{aligned}$$

where the efficiency $\epsilon ^{\upmu }_\mathrm {ID|Glo}$ is the fraction of global muons that fulfill the full set of muon identification requirements; the efficiency $\epsilon ^{\upmu }_\mathrm {Glo|Sta}$ is the global muon efficiency, given by the fraction of standalone muons that also qualify as global muon; and the efficiency $\epsilon ^{\upmu }_\mathrm {Sta|Trk}$ is the standalone muon efficiency, defined as the fraction of muons with good inner tracks that are matched within $\Delta R<0.3$ to outer standalone muon tracks with $p_{\textrm{T}} >20\,\text {Ge\hspace{-.08em}V}$ and $|\eta | <2.4$. To obtain an unbiased set of inner tracks for the measurement of the efficiency $\epsilon ^{\upmu }_\mathrm {Sta|Trk}$, inner tracks that are seeded from the extrapolation of outer standalone muon tracks are excluded. The term $c_{\mathrm {T \& P}}$ accounts for the correlations between the efficiency terms in Eq. (6). The pileup dependence of the correction from $c_{\mathrm {T \& P}}$ between the $\textrm{lowPU}$ and the $\textrm{highPU}$ data sets is estimated from simulation to be about 0.01%.

The efficiencies are determined from the data using a “tag-and-probe” methodology [1]. Identified muon candidates that are matched to the HLT muon are selected as “tag”. For each tag, a probe muon candidate of opposite charge is selected under the condition that the muon candidate pair has an invariant mass between 60 and 120$\,\text {Ge\hspace{-.08em}V}$. The efficiency $\epsilon ^{\upmu }_{x|y}$ is then measured as

7 $$\begin{aligned} \epsilon ^{\upmu }_{x|y} = \frac{n^\textrm{p}}{n^\textrm{p} + n^\textrm{f}}, \end{aligned}$$

where *y* denotes the reference sample of muon candidates and *x* is the probe criterion. The numbers $n^\textrm{p}$ and $n^\textrm{f}$ correspond to the number of events that pass and fail the test criterion, respectively.

For each of the efficiencies, and in bins of 20$\,\text {pb}^{-1}$, fits to the $m_{\upmu \upmu }$ distributions of the passing and failing distributions are performed. In the fits, the same shapes as described in Sect. 3.1 are used to describe the signal. In the histograms with passing probes, the background contribution is low and a falling exponential is used. In the case of failing probes, the nonresonant background is much larger and a more complex analytic function, comprising an exponential at high mass above the Z boson resonance and an error function at low mass, is fit. To ensure a bias-free measurement of $\epsilon ^{\upmu }_\mathrm {Glo|Sta}$, the outer standalone muon track parameters are used to determine $m_{\upmu \upmu }$ for the passing and failing probes. Since the resolution of these tracks is much worse, the invariant mass requirement is widened to 50–130$\,\text {Ge\hspace{-.08em}V}$. In the case that, in a given event, the probe muon also fulfills the tag muon requirements, the tag-and-probe muons are indistinguishable and both muons are used as probes. Quantitative results for the measurement of the efficiencies are presented in Sect. 4.

### Acceptance correction

To determine the true number of Z bosons in the visible phase space, an acceptance correction for losses, or gains, due to the finite resolution of the reconstructed muon tracks is required. The correction affects the number of reconstructed Z bosons itself. The efficiencies are also affected, primarily in the matching of inner and outer tracks, and, to a lesser extent, if muon tracks for passing and for failing probes have different resolutions. The size of the correction is determined from the simulation by comparing the efficiency-corrected number of Z bosons as obtained from the measurement with the generated number of Z bosons in the visible phase space, as defined for bare leptons after final-state radiation (FSR), but before detector simulation.

For outer muon tracks, resolution effects lead to an acceptance correction of about 1.35%, which is independent of pileup and constant over the full year of data taking. For inner tracks, the acceptance correction is 0.15% at low pileup, and it is negligibly small for the $\textrm{highPU}$ data set. This pileup dependency of 0.15% is applied as an additional correction, and an uncertainty of 100% of the correction is assigned. For a direct cross section measurement, the size of the bias could be further reduced through optimized track selection criteria. However, for this analysis it suffices that the pileup-independent components of the acceptance correction cancel in the cross section ratio.

**Fig. 3:**
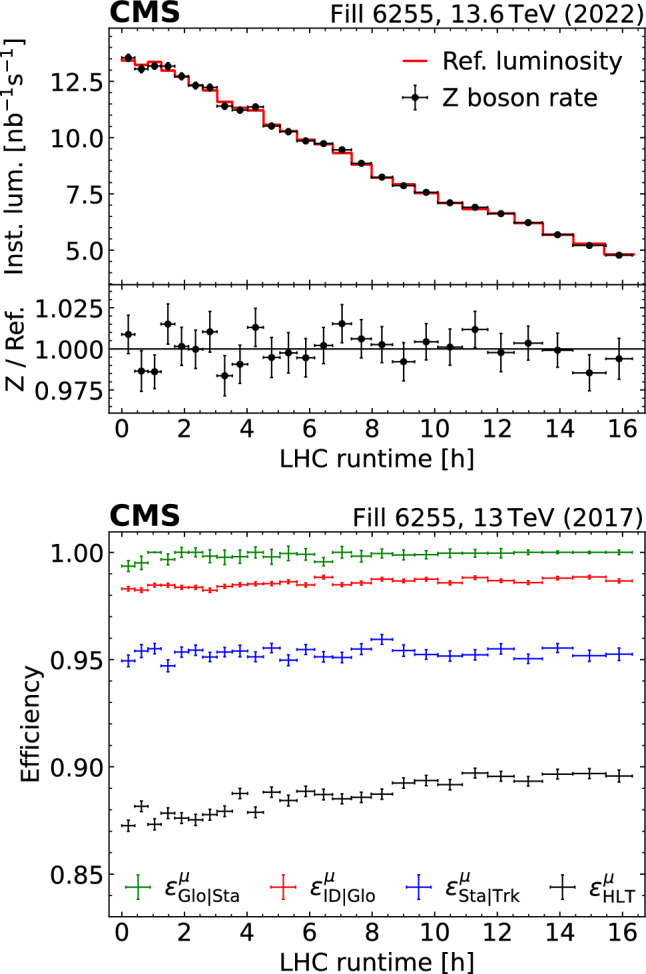
Upper: the efficiency-corrected Z boson rate, compared to the reference luminosity measurement, in the LHC fill 6255, recorded on September 29, 2017 [33]. Each bin corresponds to about 20$\,\text {pb}^{-1}$, as determined by the reference measurement. For shape comparison, the integrated Z boson rate is normalized to the reference integrated luminosity. The panel at the bottom shows the ratio of the two measurements. The vertical error bars show the statistical uncertainty in the Z boson rate. Lower: the measured single-muon efficiencies as functions of time for the same LHC fill. The vertical error bars show the statistical uncertainty in the efficiency

### The L1 trigger corrections

The term “prefiring” describes the effect that a trigger decision is assigned to a bunch crossing preceding the one in which the collision actually took place. In the CMS experiment, the triggering and readout of events in adjacent bunch crossings is vetoed in the trigger logic. However, due to the limited time resolution of the muon system, the assignment of muon candidates to bunch crossings can be wrong, and thus lead to a loss of good events, i.e., a trigger inefficiency. Since the tag-and-probe efficiency measurement is insensitive to this effect, the inefficiency due to prefiring is measured in a dedicated analysis. During the 2017 data taking, measurable prefiring occurred at nonnegligible rates for the L1 muon and ECAL triggers [42, 50]. For the L1 muon trigger, a correction for trigger inefficiency of 0.6% was found, independent of pileup and time. In contrast, losses due to prefiring of the ECAL require a pileup-dependent correction of 0.05–0.2% for the pileup range 0–50. The prefiring from ECAL triggers is caused mainly by initial or final state radiation, pileup jets, or the underlying event. The impact on the $\textrm{lowPU}$ data was somewhat larger due to the lower ECAL trigger thresholds, and for the $\textrm{lowPU}$ data a correction of 0.6% is applied.

## Results and uncertainties

The procedures described above to measure the number of reconstructed Z bosons and their efficiencies are applied to the data in bins of 20$\,\text {pb}^{-1}$. Since the amount of data at the end of a fill does not usually add up to 20$\,\text {pb}^{-1}$, the last bin is included as long as it contains more than 10$\,\text {pb}^{-1}$. In case the last bin contains ${<}10{\,\text {pb}^{-1}}$ it is merged with the second to last. Altogether, in 2017 about 2000 such bins are defined.

### Normalized Z boson rate

In Fig. 3, the measured Z boson rate and efficiencies are shown for the data recorded during a typical LHC fill of the $\textrm{highPU}$ data-taking period in 2017. In this fill, $\text {pp}$ collision data were recorded continuously for about 16 h. An integrated luminosity of about 515$\,\text {pb}^{-1}$ was accumulated, corresponding to 25 bins of 20$\,\text {pb}^{-1}$ each, and the last bin contains the remaining 15$\,\text {pb}^{-1}$ of data. The instantaneous luminosity decreased from initially 15$\,\text {nb}^{-1}\,\text {s}^{-1}$, corresponding to a pileup of about 50, to about one third of the initial value. In Fig. 3 (upper), a comparison between the conventional measurement of the recorded luminosity and the measurement using the Z boson rate is shown. The integral of the measured Z boson rate is normalized to the integral of the reference luminosity. The shapes of the two independent measurements agree very well. In Fig. 3 (lower), the muon trigger and identification efficiencies, $\epsilon ^{\upmu }_\textrm{HLT}$ and $\epsilon ^{\upmu }_\textrm{ID}$, separated into its different components, as applied to the respective time intervals, are presented. In particular, a significant dependence on time, and thus on pileup, is seen for the HLT muon efficiency for which a rise by about 3% is measured as the pileup decreases in the course of the fill.

**Fig. 4:**
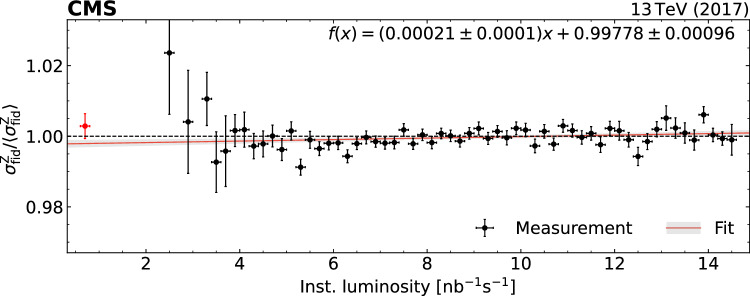
Fiducial Z boson production cross section as a function of the instantaneous recorded luminosity, normalized to the average measured cross section. In each point, multiple measurements of the delivered Z boson rates are combined, the error bars correspond to the statistical uncertainties of the Z boson rate measurement. The leftmost point, highlighted in red, corresponds to the $\textrm{lowPU}$ data. The result of a fit to a linear function is shown as a red line and the statistical uncertainties are covered by the gray band

To compare the relative linearity between the measurement of the Z  boson rate and the CMS reference luminosity, the fiducial cross section for Z boson production, normalized to the average Z  boson cross section, is studied as a function of the instantaneous luminosity. The result is shown in Fig. 4, where the average instantaneous luminosity in each 20$\,\text {pb}^{-1}$ bin is used to assign an instantaneous luminosity bin from which the average cross section is obtained. The straight-line fit to the data yields a value of 0.2% below unity for the intercept with the *y*-axis at low pileup. This value gives an estimate of the agreement between Z boson counting and reference luminosity measurement in the extrapolation from the low to the high pileup data.

### Measurement of the absolute luminosity

Using Eq. (2), the integrated luminosity of the $\textrm{highPU}$ data, referred to in the following as “Z luminosity”, is determined from the integrated luminosity of the $\textrm{lowPU}$ data, using the ratio of the number of Z bosons recorded during the two periods, corrected for reconstruction and trigger efficiencies as determined in intervals of 20$\,\text {pb}^{-1}$. In the ratio, all correlated uncertainties cancel, as detailed in the following section.

**Fig. 5:**
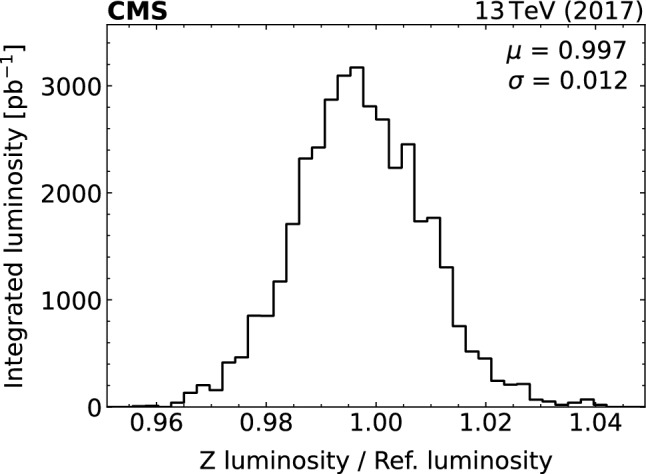
Distribution of the ratio of integrated luminosities between Z boson counting and the reference luminometer. The entries, each corresponding to one interval of 20$\,\text {pb}^{-1}$ of $\textrm{highPU}$ data, are weighted with the respective measured luminosity

**Fig. 6:**
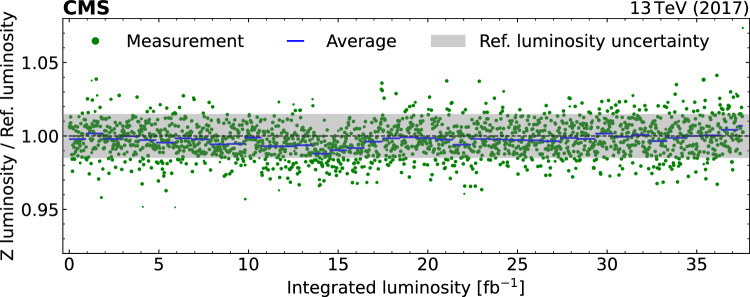
The luminosity as measured from Z bosons divided by the reference luminosity as a function of the integrated luminosity for the 2017 $\textrm{highPU}$ data. Each green point represents the ratio from one measurement of the number of Z bosons. The blue lines show the averages of 50 consecutive measurements, corresponding to an average of 1$\,\text {fb}^{-1}$ of data. The gray band has a width of 1.5%, corresponding to the uncertainty in the ratio of the integrated reference luminosities from the $\textrm{lowPU}$ to the one of $\textrm{highPU}$  [33]

In Fig. 5, the distribution of the ratios between the Z luminosity and the reference luminosity as obtained from the CMS luminosity systems is shown. Each entry in the histogram corresponds to an interval of 20$\,\text {pb}^{-1}$ in the $\textrm{highPU}$ data recorded in 2017. The central values of both measurements are in good agreement with a difference of 0.3%. The standard deviation of about 1.2% is predominantly of statistical nature, and close to the expectation for the pure statistical uncertainty of about 12 000 Z boson candidates reconstructed in intervals of 20$\,\text {pb}^{-1}$ each. The ratio of Z luminosity and reference luminosity as a function of the integrated luminosity is shown in Fig. 6. This figure shows a good stability of the Z luminosity measurement over the full year. No significant patterns in time are observed.

### Statistical and systematic uncertainties, and additional cross checks

The uncertainties in the analysis were studied with the focus on the ratio $r=N^{{\textrm{Z}}} _\textrm{highPU}/N^{{\textrm{Z}}} _\textrm{lowPU}$ of the Z boson counts between two data samples in 2017 as presented in Eq. (2). The full list of considered sources of uncertainty in the cross sections and their ratio is given in Table 1, and described in the following.

Statistical uncertainties are driven by the number of available Z  bosons and also include the statistical uncertainty in the efficiencies. As mentioned above, in one interval of 20$\,\text {pb}^{-1}$, about 12 000 Z bosons candidates with two muons in the final state are available, leading to an average statistical uncertainty of 1.17%. For all intervals combined, the statistical uncertainty for the full 2017 $\textrm{highPU}$ data is negligibly small. The $\textrm{lowPU}$ data set corresponds to an integrated luminosity of about 200$\,\text {pb}^{-1}$, and this contributes a statistical uncertainty of about 0.35%.

As discussed in Sect. 3.2, the correction factor for correlations in the trigger efficiencies of the two muons $C_{\textrm{HLT}}$ is determined from data and simulation; it is about 0.1% above unity for the $\textrm{highPU}$ sample, consistently for data and MC simulation. The uncertainty in $C_{\textrm{HLT}}$ is assigned to be 100% of the correction.

Possible correlations between the two identified muons and imperfect factorization of muon identification and reconstruction efficiencies were discussed in Sect. 3.3. The simulation shows negligible effects, and corrections at the level of 0.01% are applied. The corresponding uncertainties are estimated to be 100% of the correction.

The limited resolution of the reconstructed muon tracks leads to a bias in the measurement, as described in Sect. 3.4. The bias from the inner track resolution is smaller, but pileup dependent, and remains in the ratio with a magnitude of 0.15%. The outer track resolution leads to a large bias, but is mostly pileup independent and cancels in the ratio. A correction is derived from simulation and two independent sources of uncertainty, estimated to be 100% of the correction each, are assigned each for the inner and outer tracks, respectively.

Systematic uncertainties in the L1 muon prefiring corrections, described in Sect. 3.5, cancel completely in the ratio, whereas the uncertainties due to ECAL prefiring have a different magnitude between the two data sets and cancel only partially. The remaining uncertainty is estimated to be 20% of the nominal correction [42].

The extraction of the signal and background contributions was studied using alternative fit models. For the signal model, the Gaussian resolution function convolved with the histogram template is varied. First, the histogram template is used alone, i.e., fully relying on the simulation and leaving no further degrees of freedom to the fit. Secondly, the histogram template is convolved with a Crystal Ball function [52], which has four free parameters and gives the fit more freedom to incorporate possible differences between data and simulation. Thirdly, the histogram template is constructed from generator-level post-FSR leptons, instead of mirroring the selection at detector level. This template is then convolved with a Crystal Ball function. The three variations lead to changes in the extracted numbers of Z bosons, and the efficiencies, in both the $\textrm{highPU}$ and $\textrm{lowPU}$ data sets. While the trends are correlated, the relative magnitudes are different, and this leads to a significant residual uncertainty. The envelope of the three variations is taken to quantify this uncertainty.

The two types of background models are varied independently. For the categories with major background contributions, the Das function [53], a wide Gaussian distribution with exponential tails is used as an alternative function, which has four free parameters, as opposed to three for the nominal model. In the other cases, the falling exponential is substituted by a uniform distribution.

**Table 1:** Summary of the uncertainties in the number of delivered Z bosons in the 2017 $\textrm{highPU}$ and $\textrm{lowPU}$ data, and their ratio. The symbol $\delta$ denotes the relative uncertainty, i.e., $\delta x = \Delta x / x$. The systematic and statistical uncertainties are added in quadrature to obtain the total uncertainty

	$\delta N^{{\textrm{Z}}} _\textrm{highPU}$ [%]	$\delta N^{{\textrm{Z}}} _\textrm{lowPU}$ [%]	$\delta \Big (N^{{\textrm{Z}}} _\textrm{highPU}/N^{{\textrm{Z}}} _\textrm{lowPU} \Big )$ [%]
HLT correlation $C_{\textrm{HLT}}$	$\pm 0.1$	$\pm 0.06$	$\pm 0.04$
Dimuon correlation $C_{\textrm{ID}}$	$\pm 0.00$	${\mp } 0.01$	$\pm 0.01$
Inner-outer track correlation $c_{\mathrm {T \& P}}$	$\pm 0.01$	${\mp } 0.01$	$\pm 0.01$
Inner track resolution	$\pm 0.01$	$\pm 0.16$	${\mp } 0.15$
Outer track resolution	$\pm 1.35$	$\pm 1.36$	${\mp } 0.01$
L1 muon prefiring	$\pm 0.15$	$\pm 0.15$	0
ECAL prefiring	$\pm 0.04$	$\pm 0.14$	${\mp } 0.10$
Signal modeling up	$-0.63$	$-0.75$	$+0.19$
Signal modeling down	$+0.51$	$+0.71$	$-0.21$
Background modeling up	$-0.15$	$-0.31$	$+0.16$
Background modeling down	$-0.09$	$-0.05$	$-0.04$
Systematic up	$+1.45$	$+1.56$	$+0.31$
Systematic down	$-1.50$	$-1.60$	$-0.28$
Statistical	$\pm 0.03$	$\pm 0.35$	$\pm 0.35$
Total up	$+1.45$	$+1.60$	$+0.47$
Total down	$-1.50$	$-1.64$	$-0.45$

The total systematic uncertainty is obtained by adding the systematic uncertainties listed in Table 1 in quadrature. In combination with the statistical uncertainty of 0.35%, the total uncertainty to transfer the luminosity from the $\textrm{lowPU}$ data to the $\textrm{highPU}$ data in 2017 is

8 $$\begin{aligned} \delta r = {}^{+0.31\%}_{-0.28\%}\,\text {(syst)} \pm 0.35\%\,\text {(stat)} = {}^{+0.47\%}_{-0.45\%}\,, \end{aligned}$$

where the statistical uncertainty is due to the limited size of the $\textrm{lowPU}$ data set. The systematic uncertainty is driven by the uncertainties in the signal modeling, followed by the background modeling and acceptance corrections. Overall, a total uncertainty of about 0.5% is obtained.

Multiple cross-checks were performed to test the robustness of the result. The size of the luminosity bin was varied from 20 down to 15 and up to 30$\,\text {pb}^{-1}$, and negligible differences with respect to the nominal measurement were found. It was further verified that the measurement is independent of the choice of the bin width chosen for the $m_{\upmu \upmu }$ distribution, by varying it by factors of 1/2 and 2. Independence of the result on the chosen fit interval was tested using two alternative ranges: a more narrow interval from $m_{\upmu \upmu } \in [70,110]\,\text {Ge\hspace{-.08em}V}$ and a wider interval from $m_{\upmu \upmu } \in [50,130]\,\text {Ge\hspace{-.08em}V}$. Both variations have a strong impact on $N^{{\textrm{Z}}}$ since the phase space of the measurement changes, but, as expected, the effect cancels almost completely in the ratio. The results of these cross checks are summarized in Table 2.

**Table 2:** Summary of cross checks performed by varying the length of the luminosity interval, the bin width of the $m_{\upmu \upmu }$ histograms, and the range of the fit. As in Table 1, the resulting variations of the number of Z bosons in the 2017 $\textrm{highPU}$ and $\textrm{lowPU}$ data, and their ratio, are shown. The $\delta$ denotes the relative variations, i.e., $\delta x = \Delta x / x$

	$\delta N^{{\textrm{Z}}} _\textrm{highPU}$ [%]	$\delta N^{{\textrm{Z}}} _\textrm{lowPU}$ [%]	$\delta \Big (N^{{\textrm{Z}}} _\textrm{highPU}/N^{{\textrm{Z}}} _\textrm{lowPU} \Big )$ [%]
Lum. bin size 30$\,\text {pb}^{-1}$	$-0.05$	$-0.01$	$-0.04$
Lum. bin size 15$\,\text {pb}^{-1}$	$+0.04$	$+0.07$	$-0.03$
Mass bin width 1$\,\text {Ge\hspace{-.08em}V}$	$-0.02$	$-0.06$	$+0.03$
Mass bin width 0.25$\,\text {Ge\hspace{-.08em}V}$	$-0.01$	$+0.01$	$-0.02$
Mass range $[50,130]\,\text {Ge\hspace{-.08em}V}$	$+1.25$	$+1.24$	$+0.00$
Mass range $[70,110]\,\text {Ge\hspace{-.08em}V}$	$-2.32$	$-2.26$	$-0.05$

## Discussion and outlook

With an uncertainty in the transfer factor of about 0.5% for the 2017 data, this analysis shows that Z boson counting can provide an independent and competitive method to extrapolate and integrate luminosity calibrations. The results from Z boson counting are independent of the conventional luminosity measurements. They can be treated as uncorrelated in combinations, which can lead to significant improvements in the combined uncertainty.

Taking the current precision of 1.7% for the integrated luminosity in the $\textrm{lowPU}$ data [33], the integrated luminosity in the $\textrm{highPU}$ 2017 data could potentially be determined to a precision of better than 1.8%, in contrast to the preliminary uncertainty of the reference luminosity measurement of 2.3% [33].

A unique aspect of Z boson counting is that the relevant efficiency corrections as a function of time can be calibrated from the same event sample. This feature makes the method robust not only against small changes in detector response, but also across different detector configurations. In general, once a precision measurement of the integrated luminosity is available, such as that for the $\textrm{lowPU}$ data in 2017, the integrated luminosity for all data recorded at the same center-of-mass energy can be determined using the Z boson counting. However, each transfer between data sets requires detailed studies of the correlations of the muon trigger and the reconstruction efficiencies.

In this paper, the full analysis was presented for the data from 2017, when a dedicated and sufficiently large sample of $\textrm{lowPU}$ data was recorded. Under such conditions, a large fraction of the systematic uncertainties cancels in the ratio. For the most precise CMS measurement of the luminosity to date [29], published for 2016, an extrapolation and integration uncertainty of 0.7% was reported. For 2016, no $\textrm{lowPU}$ data set was recorded. Further studies on the impact of different detector conditions would be required to extrapolate from the 2016 data set. If, hypothetically, an extrapolation uncertainty of 0.5% for Z boson counting were achievable also in the 2016 data, the uncertainty of 1.2% in the total integrated luminosity for 2016 could be improved to 1.1%.

The dominant contribution to the uncertainty comes from the statistical uncertainty, which is driven by the size of the $\textrm{lowPU}$ data sample. The $\textrm{lowPU}$ data recorded in 2017 correspond to an integrated luminosity of about 200$\,\text {pb}^{-1}$. A significant increase of the sample size, e.g., by a factor 3 or 4, would make the statistical uncertainty negligible.

In the coming years, during the ongoing LHC Run 3 and beyond, additional measurements and studies on the main systematic uncertainties will be performed, and that is expected to improve the precision of the method further. Furthermore, the method is expected to contribute substantially to the combination of integrated luminosity measurements for different data sets. In the longer term, pileup conditions of up to 200 $\text {pp}$ collisions per bunch crossing are expected at the HL-LHC [31]. In both Run 3 and at the HL-LHC, the uncertainties due to extrapolation from vdM conditions to standard data taking are expected to remain substantial. In such conditions, the method of Z boson counting has the potential to provide significant improvements.

## Summary

The precision measurement of the Z boson production rate provides a complementary method to transfer integrated luminosity measurements between data sets. This study makes use of events with Z bosons decaying into a pair of muons. The data were recorded with the CMS experiment at the CERN LHC in 2017, at a proton–proton center-of-mass energy of 13$\,\text {Te\hspace{-.08em}V}$. The integrated luminosity of a larger data sample recorded in 2017 is obtained from that of a smaller data set recorded at lower pileup using the ratio of the efficiency-corrected numbers of Z bosons counted in the two data sets. The full set of efficiencies and correlation correction factors for triggering, reconstruction, and selection are determined in intervals of 20$\,\text {pb}^{-1}$ from the same Z boson data samples. Monte Carlo simulations are used only to describe the shape of the resonant Z boson signal and for the study of possible biases of the method. A detailed quantitative study of the systematic uncertainties and their dependencies on pileup is performed for the first time. In the integrated luminosity ratio, the systematic uncertainties cancel almost completely, with the exception of the pileup-dependent effects. The resulting uncertainty in the ratio is 0.5%. With its high precision, the Z boson counting is competitive with and independent of conventional methods for the extrapolation and integration of luminosity.

## Data Availability

This manuscript has no associated data or the data will not be deposited. [Authors’ comment: Release and preservation of data used by the CMS Collaboration as the basis for publications is guided by the CMS policy as stated in https://cms-docdb.cern.ch/cgibin/PublicDocDB/RetrieveFile?docid=6032 &filename=CMSDataPolicyV1.2.pdf &version=2. CMS data preservation, re-use and open access policy.]
